# Suppression of a Natural Killer Cell Response by Simian Immunodeficiency Virus Peptides

**DOI:** 10.1371/journal.ppat.1005145

**Published:** 2015-09-02

**Authors:** Jamie L. Schafer, Moritz Ries, Natasha Guha, Michelle Connole, Arnaud D. Colantonio, Emmanuel J. Wiertz, Nancy A. Wilson, Amitinder Kaur, David T. Evans

**Affiliations:** 1 Department of Microbiology and Immunobiology, Harvard Medical School, New England Primate Research Center, Southborough, Massachusetts, United States of America; 2 Department of Pathology and Laboratory Medicine, University of Wisconsin, Madison, Wisconsin, United States of America; 3 Division of Immunology, Harvard Medical School, New England Primate Research Center, Southborough, Massachusetts, United States of America; 4 Department of Medical Microbiology, University Medical Center Utrecht, Utrecht, The Netherlands; Emory University, UNITED STATES

## Abstract

Natural killer (NK) cell responses in primates are regulated in part through interactions between two highly polymorphic molecules, the killer-cell immunoglobulin-like receptors (KIRs) on NK cells and their major histocompatibility complex (MHC) class I ligands on target cells. We previously reported that the binding of a common MHC class I molecule in the rhesus macaque, Mamu-A1*002, to the inhibitory receptor Mamu-KIR3DL05 is stabilized by certain simian immunodeficiency virus (SIV) peptides, but not by others. Here we investigated the functional implications of these interactions by testing SIV peptides bound by Mamu-A1*002 for the ability to modulate Mamu-KIR3DL05^+^ NK cell responses. Twenty-eight of 75 SIV peptides bound by Mamu-A1*002 suppressed the cytolytic activity of primary Mamu-KIR3DL05^+^ NK cells, including three immunodominant CD8^+^ T cell epitopes previously shown to stabilize Mamu-A1*002 tetramer binding to Mamu-KIR3DL05. Substitutions at C-terminal positions changed inhibitory peptides into disinhibitory peptides, and vice versa, without altering binding to Mamu-A1*002. The functional effects of these peptide variants on NK cell responses also corresponded to their effects on Mamu-A1*002 tetramer binding to Mamu-KIR3DL05. In assays with mixtures of inhibitory and disinhibitory peptides, low concentrations of inhibitory peptides dominated to suppress NK cell responses. Consistent with the inhibition of Mamu-KIR3DL05^+^ NK cells by viral epitopes presented by Mamu-A1*002, SIV replication was significantly higher in Mamu-A1*002^+^ CD4^+^ lymphocytes co-cultured with Mamu-KIR3DL05^+^ NK cells than with Mamu-KIR3DL05^-^ NK cells. These results demonstrate that viral peptides can differentially affect NK cell responses by modulating MHC class I interactions with inhibitory KIRs, and provide a mechanism by which immunodeficiency viruses may evade NK cell responses.

## Author Summary

Natural killer (NK) cells recognize and kill infected cells without prior antigenic stimulation, and thus provide an important early defense against virus infection. NK cell responses in primates are regulated in part through interactions between two highly polymorphic molecules, the killer-cell immunoglobulin-like receptors (KIRs) on NK cells and their major histocompatibility complex (MHC) class I ligands on target cells. Inhibitory KIRs normally suppress NK cell responses through interactions with their MHC class I ligands on the surface of healthy cells. However, when these interactions are perturbed, this inhibition is lost resulting in NK cell activation and killing of the target cell. We investigated the functional implications of simian immunodeficiency virus (SIV) peptides bound by a common MHC class I molecule in the rhesus macaque that stabilize or disrupt binding to an inhibitory KIR. Whereas SIV peptides that stabilized KIR-MHC class I binding suppressed NK cell activation, peptides that disrupted this interaction did not and resulted in NK cell lysis. These findings demonstrate that viral peptides can modulate NK cell responses through KIR-MHC class I interactions, and are consistent with the possibility that human and simian immunodeficiency viruses may acquire changes in epitopes that increase the binding of MHC class I ligands to inhibitory KIRs as a mechanism to suppress NK cell responses.

## Introduction

By virtue of their ability to recognize and kill infected cells without prior exposure to antigen, natural killer (NK) cells provide an important innate defense against viral pathogens. NK cells differentiate virus-infected cells from healthy cells through the integration of complex signals from activating and inhibitory receptors, which in primates include the highly polymorphic killer-cell immunoglobulin-like receptors (KIRs). Whereas the molecular basis of ligand recognition for the activating KIRs is not fully understood, inhibitory KIRs selectively bind to subsets of major histocompatibility complex (MHC) class I molecules bearing particular sequence motifs in their α1-domains [1–3]. Inhibitory KIRs normally suppress NK cell activation through interactions with their MHC class I ligands on the surface of healthy cells. However, when these interactions are perturbed, for instance as a result of MHC class I downregulation by the human immunodeficiency virus (HIV)-1 Nef protein [4–6], this inhibition is lost resulting in NK cell degranulation and lysis of the infected cell.

Polymorphic differences in *KIR* and *HLA class I* genes can influence the course of HIV-1 infection [7–12], as well as the outcome of infection with other viral pathogens, including hepatitis C virus (HCV) [13], human papillomavirus (HPV) [14] and cytomegalovirus (CMV) [15]. In the case of HIV-1, activating and highly-expressed inhibitory alleles of *KIR3DL1/S1*, in combination with *HLA-Bw4* alleles encoding isoleucine at position 80 (HLA-Bw4-80I), are associated with delayed progression to AIDS and greater suppression of viral replication in autologous CD4^+^ T cells by bulk NK cells [8,9,16]. In accordance with these genetic associations, NK cells expressing KIR3DS1 can suppress the *in vitro* replication of HIV-1 in HLA-Bw4-80I^+^ lymphocytes [17], and KIR3DS1^+^ and KIR3DL1^+^ NK cells preferentially expand during HIV-1 infection in HLA-Bw4-80I^+^ individuals [18]. KIR-expressing NK cells may also exert selective pressure on virus replication as demonstrated by HIV-1 polymorphisms associated with KIR2DL2 that confer resistance to NK cells expressing this KIR [19].

Consistent with three-dimensional structures of KIR-HLA class I complexes revealing that KIRs contact surfaces of their HLA class I ligands over C-terminal residues of the bound peptide [20–22], changes in C-terminal positions of the peptide can stabilize or disrupt interactions with inhibitory KIRs [23–26]. NK cells may therefore sense changes in the repertoire of peptides presented by the MHC class I ligands of inhibitory KIRs. Although this helps to explain how NK cells differentiate virus-infected cells from healthy cells, it also creates an opportunity for immune escape. By acquiring changes in epitopes that enhance MHC class I binding to inhibitory KIRs, viruses may prevent the elimination of infected cells by NK cells. Indeed, such a mechanism may account for the adaptation of HIV-1 for resistance to KIR2DL2^+^ NK cells [19].

We previously demonstrated that the binding of Mamu-A1*002, a common MHC class I molecule in the rhesus macaque, to Mamu-KIR3DL05 is stabilized by certain simian immunodeficiency virus (SIV) peptides, but not by others [27]. To investigate the functional implications of these interactions, we tested SIV peptides bound by Mamu-A1*002 for the ability to suppress the cytolytic activity of primary Mamu-KIR3DL05^+^ NK cells. Twenty-eight of the 75 peptides, including three immunodominant CD8^+^ T cell epitopes with the highest affinity for Mamu-A1*002, inhibited killing by Mamu-KIR3DL05^+^ NK cells. The inhibitory effects of these peptides were dominant over variants that did not suppress NK cell killing. Moreover, Mamu-KIR3DL05^+^ NK cells were impaired for the ability to suppress SIV replication in Mamu-A1*002^+^, but not in Mamu-A1*002^-^, lymphocytes. These observations demonstrate that viral peptides can modulate NK cell activity and are consistent with the possibility that HIV-1 and SIV may acquire changes in epitopes that increase the binding of MHC class I ligands to inhibitory KIRs to impair NK cell killing of virus-infected cells.

## Results

### Mamu-A1*002-SIV peptide complexes differentially inhibit Mamu-KIR3DL05^+^ NK cells

We previously reported that Mamu-A1*002 tetramers folded with some SIV peptides, but not others, bind to Mamu-KIR3DL05 [27]. To evaluate the functional implications of these interactions, we tested individual peptides for the ability to inhibit NK cell degranulation in cytotoxicity assays. Primary NK cells were expanded from rhesus macaque peripheral blood mononuclear cells (PBMCs) by stimulation with γ-irradiated K562 Clone9.mbIL21 cells [28] and sorted into Mamu-KIR3DL05^+^ and -KIR3DL05^-^ subsets using Mamu-A1*002 Gag GY9 tetramers. These NK cells were then tested in cytoxicity assays for recognition of a Mamu-A1*002-expressing, transporter associated with antigen processing (TAP)-deficient 721.221 cell line (721.221-ICP47-A1*002) incubated with specific SIV peptides. Because TAP is necessary for the translocation of peptides from the cytoplasm into the endoplasmic reticulum, MHC class I molecules expressed in these cells cannot load peptides derived from endogenously synthesized proteins, and are rapidly internalized from the plasma membrane [29,30]. Thus, most of the Mamu-A1*002 molecules expressed by these cells are empty and can be loaded with exogenous peptides. Moreover, peptide binding to Mamu-A1*002 can be assessed by staining for an increase in steady-state levels of MHC class I expression on the cell surface.

**Fig 1:**
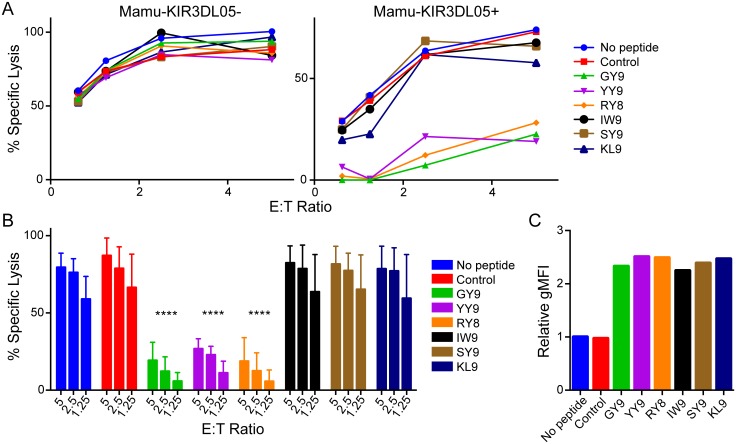
Peptide-dependent inhibition of Mamu-KIR3DL05^+^ NK cells. (A) Mamu-KIR3DL05^+^ and -KIR3DL05^-^ NK cells from the same animal were incubated with CAM-labeled 721.221-ICP47-A1*002 target cells pulsed with the indicated SIVmac239 peptides. Percent specific lysis was calculated from the amount of CAM released into the culture supernatant after 4 hours at the indicated effector to target (E:T) ratios. The results shown are representative of data obtained with NK cells from three different animals. Controls include target cells incubated without peptide (No peptide) or with a GY9 variant with substitutions at anchor positions (S2A & Y9G) that abrogate binding to Mamu-A1*002 (Control). (B) Bar graphs summarize the mean percent specific lysis for independent experiments with Mamu-KIR3DL05^+^ NK cells from three different animals. Error bars indicate +1 standard deviation (SD) and asterisks indicate significant differences in the lysis of target cells pulsed with each SIV peptide compared to control cells without peptide (****p<0.001 by two-way ANOVA with Dunnett’s test). (C) Stabilization of Mamu-A1*002 on the surface of 721.221-ICP47-A1*002 cells was determined by staining with the pan-MHC class I monoclonal antibody W6/32 and the relative geometric mean fluorescence intensity (gMFI) normalized to cells incubated without peptide is shown. Data is representative of three independent experiments.

Six CD8^+^ T cell epitopes of SIV bound by Mamu-A1*002 were initially tested for their ability to suppress the cytolytic activity of Mamu-KIR3DL05^+^ NK cells. These included Gag_71-79_ GY9, Nef_159-167_ YY9, Env_788-795_ RY8, Vif_89-97_ IW9, Env_760-768_ SY9, and Nef_169-177_ KL9 peptides (Table 1). Mamu-KIR3DL05^-^ NK cells killed 721.221-ICP47-A1*002 cells incubated with each of these peptides to a similar extent. In contrast, the cytolytic activity of Mamu-KIR3DL05^+^ NK cells was selectively inhibited by Gag GY9, Nef YY9, and Env RY8, but not by Vif IW9, Env SY9, or Nef KL9 (Fig 1A). The same pattern of peptide-dependent inhibition was reproducible with Mamu-KIR3DL05^+^ NK cells from four different animals (Fig 1B), and was not the result of differences in MHC class I binding, as all of the peptides stabilized surface expression of Mamu-A1*002 on the target cells to similar levels (Fig 1C). These results are consistent with previously published data showing that Mamu-A1*002 tetramers folded with Gag GY9, Nef YY9 and Env RY8, but not Vif IW9, bind to Mamu-KIR3DL05 [27].

**Table 1:** SIVmac239 peptides bound by Mamu-A1*002.

Name	Position^1^	Sequence	Binding Affinity (IC_50_ nM)^2^
Nef YY9(159)	159	YTSGPGIRY	2.7
Env RY8	788	RTLLSRVY	3.1
Env VF9	322	VTIMSGLVF	4.8
Gag GY9	71	GSENLKSLY	4.9
Pol YF10	370	YTMRHVLEPF	5.5
Vif IW9	89	ITWYSKNFW	5.5
Nef KL9	169	KTFGWLWKL	6.3
Nef RM11	109	RTMSYKLAIDM	6.8
Env RY10	296	RTIISLNKYY	8.1
Nef YY9(221)	221	YTYEAYVRY	8.5
Pol LM10	497	LTEEVQWTEM	8.6
Vpr LY9	40	LTALGNHIY	9.1
Env LF11	820	LTYLQYGWSYF	9.5
Env RY9(817)	817	RTELTYLQY	9.5
Env RY9(296)	296	RTIISLNKY	10
Vpr LM10	91	LSAIPPSRSM	10
Env MY9	482	MSAEVAELY	11
Env IY11	480	ITMSAEVAELY	12
Env GY9	279	GTRAENRTY	13
Env GY11	279	GTRAENRTYIY	13
Env KM9	317	KTVLPVTIM	14
Nef LM9	248	LTARGLLNM	14
Env SY9	760	SSWPWQIEY	16
Nef RI9	109	RTMSYKLAI	16
Vif WY8	97	WTDVTPNY	17
Nef MM9	111	MSYKLAIDM	20
Env AY9	685	ASWIKYIQY	23
Env WY10	392	WTNCRGEFLY	35
Vif WF8	52	WTCSRVIF	37
Env LY10	820	LTYLQYGWSY	41
Env FF8	725	FSSPPSYF	45
Pol LF10	199	LTALGMSLNF	49
Pol WY9	503	WTEMAEAEY	51
Vif SY10	83	STYAVRITWY	51
Vif WL10	52	WTCSRVIFPL	56
Tat LA10	40	LSQLYRPLEA	57
Pol SF10	625	STPPLVRLVF	58
Nef LY9	20	LLRARGETY	65
Env QY10	590	QTRVTAIEKY	68
Env GF10	519	GTSRNKRGVF	70
Vif KY10	32	KTKDLQKVCY	71
Nef YL9	134	YSARRHRIL	73
Vif HF8	110	HSTYFPCF	73
Vpr SM9	92	SAIPPSRSM	73
Gag LY8	177	LSEGCTPY	77
Env WF8	392	WTNCRGEF	79
Nef KV10	169	KTFGWLWKLV	81
Env LY11	15	LSVYGIYCTLY	83
Vif SW9	83	STYAVRITW	83
Pol VF10	759	VSQGIRQVLF	86
Vif SA9	111	STYFPCFTA	86
Nef TY8	160	TSGPGIRY	87
Pol LY10	518	LSQEQEGCYY	87
Vif LY11(82)	82	LSTYAVRITWY	89
Pol FF9	324	FSIPLDEEF	92
Env NY10	759	NSSWPWQIEY	97
Vif LY11(75)	75	LTPEKGWLSTY	106
Env CF8	22	CTLYVTVF	110
Pol IF11	624	ISTPPLVRLVF	112
Pol LY9	518	LSQEQEGCY	124
Gag QM10	309	QTDAAVKNWM	130
Nef TM10	110	TMSYKLAIDM	131
Vif IF8	89	ITWYSKNF	136
Vpr RM9	63	RILQRALFM	157
Env QY9	359	QTIVKHPRY	189
Env VM8	260	VSSCTRMM	191
Nef RY10	19	RLLRARGETY	191
Vif YV9	92	YSKNFWTDV	241
Pol WF10	46	WSMGKEAPQF	244
Env LM8	104	LSPLCITM	331
Nef KY9	105	KVPLRTMSY	363
Env SM10	135	STTASAKVDM	373
Nef LY11	50	LSSLSCEGQKY	401
Vif YY10	104	YADILLHSTY	442
Tat RA10	104	RTRHCQPEKA	474

^1^Position indicates the location of the peptide N-terminal residue within the respective SIVmac239 protein.

^2^Relative binding affinities of SIV peptides for Mamu-A1*002 reported by Loffredo et al. [31].

**Fig 2:**
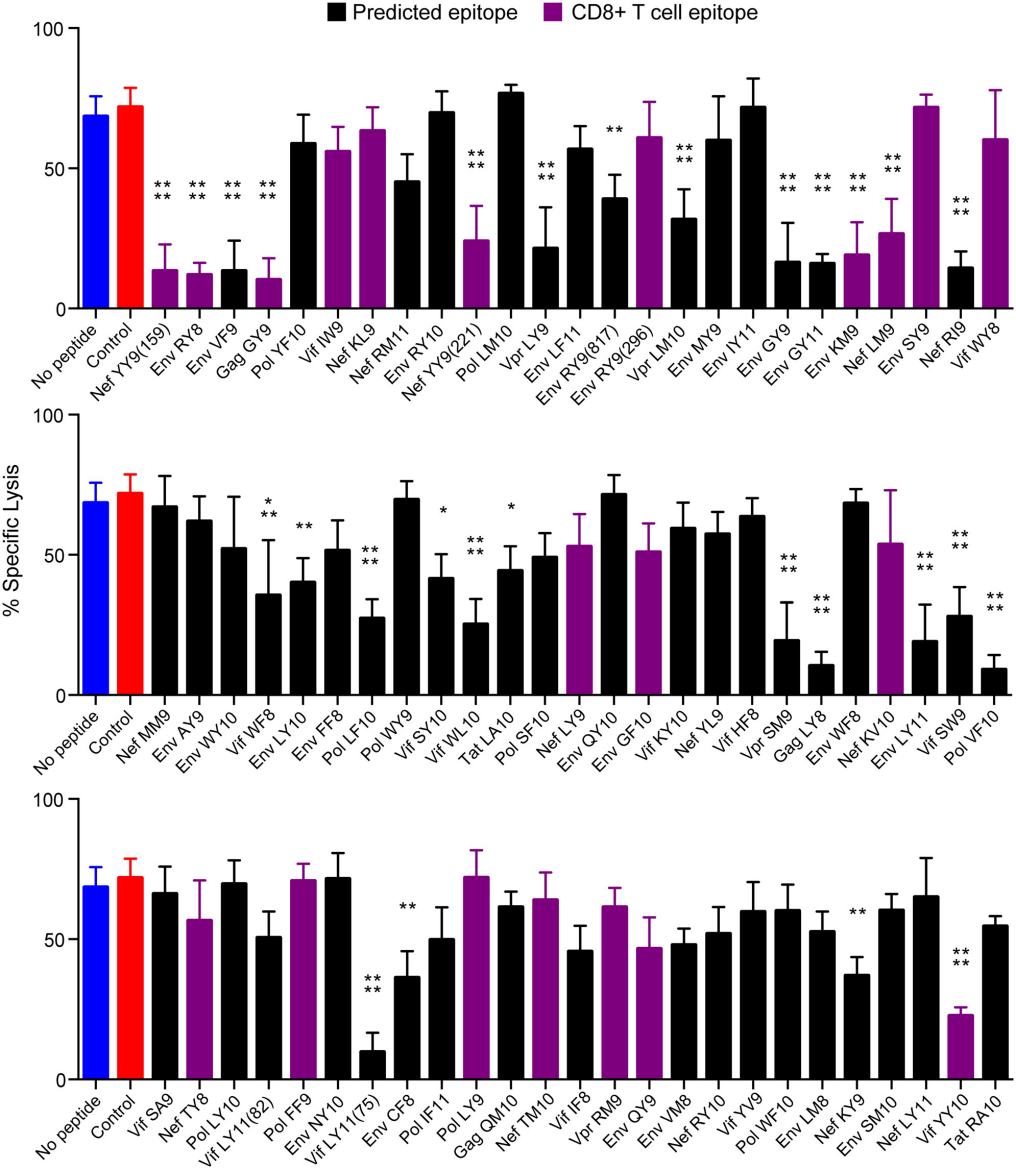
Twenty-eight of 75 SIV peptides bound by Mamu-A1*002 inhibit Mamu-KIR3DL05^+^ NK cells. Mamu-KIR3DL05^+^ NK cells were incubated at a 5:1 E:T ratio with CAM-labeled 721.221-ICP47-A1*002 target cells pulsed with the indicated SIVmac239 peptides. Percent specific lysis was calculated from the amount of CAM released into the culture supernatant after a 4-hour incubation. Bars represent the mean percent specific lysis for experiments using NK cells from three different animals. Peptides are ordered from highest to lowest affinity for Mamu-A1*002 according to Loffredo et al. [31]. Previously defined CD8^+^ T cell epitopes are indicated by purple bars and controls include target cells incubated without peptide (blue) or with a GY9 variant with substitutions at anchor positions that abrogate binding to Mamu-A1*002 (red). Error bars indicate +1 SD and asterisks indicate significant differences in the lysis of target cells pulsed with each SIV peptide compared to control cells without peptide (*p<0.05, **p<0.01, ***p<0.005, ****p<0.001 by one-way ANOVA with Dunnett’s test). Stabilization of Mamu-A1*002 on the surface of 721.221-ICP47-A1*002 cells was verified by flow cytometry using the MHC class I-specific monoclonal antibody W6/32 (S1 Fig).

The same approach was used to screen 75 SIV_mac_239 peptides previously shown to bind to Mamu-A1*002 (Table 1) [31]. As expected, all of the peptides stabilized Mamu-A1*002 expression on the surface of 721.221-ICP47-A1*002 cells (S1 Fig) and had no effect on the cytolytic activity of Mamu-KIR3DL05^-^ NK cells (S2 Fig). However, 28 of the 75 peptides significantly inhibited killing by Mamu-KIR3DL05^+^ NK cells (Fig 2). Ordered from highest to lowest binding affinity for Mamu-A1*002, these inhibitory peptides include seven previously defined CD8^+^ T cell epitopes known to be presented on the surface of SIV-infected cells [31]. Notably, the four peptides with highest affinity for Mamu-A1*002, including three immunodominant CD8^+^ T cell epitopes (Nef YY9, Env RY8, and Gag GY9; purple), strongly suppressed the cytolytic activity of Mamu-KIR3DL05^+^ NK cells (Fig 2). Because of their high affinity for Mamu-A1*002, these peptides may be presented at a greater density on the surface of SIV-infected cells than other viral peptides, and thus have a greater impact on Mamu-KIR3DL05^+^ NK cell responses.

### Substitutions in C-terminal positions of Mamu-A1*002-bound peptides affect interactions with Mamu-KIR3DL05

**Fig 3:**
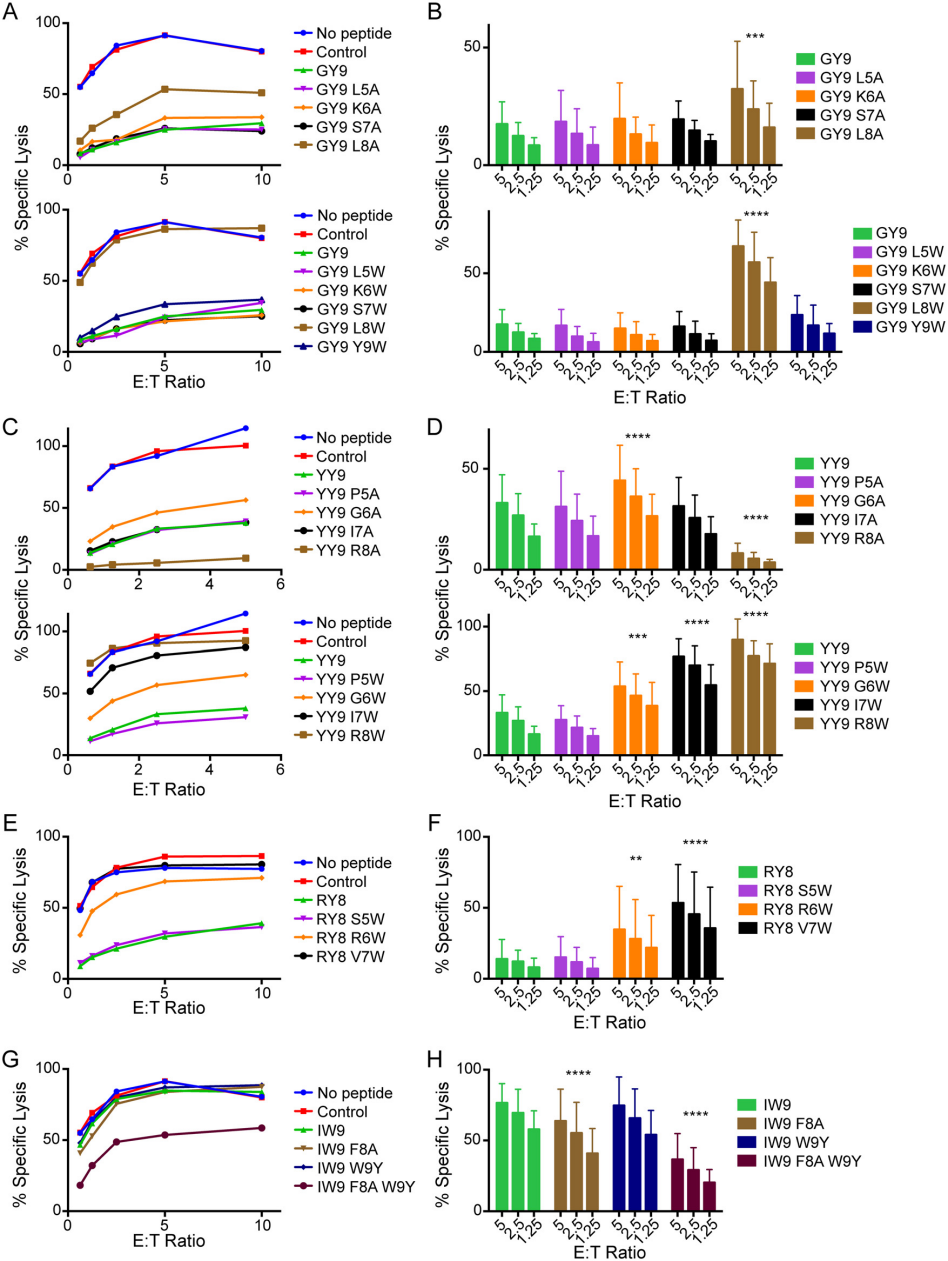
Substitutions at C-terminal positions of SIV peptides bound by Mamu-A1*002 modulate Mamu-KIR3DL05^+^ NK cell activity. Mamu-KIR3DL05^+^ NK cells were incubated with CAM-labeled 721.221-ICP47-A1*002 cells pulsed with variants of Gag GY9 (A, B), Nef YY9 (C, D), Env RY8 (E, F), and Vif IW9 (G, H), and target cell lysis was assessed after 4 hours at the indicated E:T ratios. Representative data (A, C, E & G) and mean percent specific lysis (B, D, F & H) are shown for independent experiments using NK cells from three different animals. Error bars indicate +1 SD and asterisks indicate significant differences in the lysis of target cells pulsed with wild-type SIV peptides compared to target cells pulsed with specific peptide variants (*p<0.05, ***p<0.005, ****p<0.001 by two-way ANOVA with Dunnett’s test). Mamu-A1*002 stabilization on the surface of 721.221-ICP47-A1*002 cells was verified by flow cytometry using the MHC class I-specific monoclonal antibody W6/32 (S3 Fig).

To define the residues of Mamu-A1*002-bound peptides that differentially affect interactions with Mamu-KIR3DL05, variants of Gag GY9, Nef YY9, Env RY8 and Vif IW9 were screened for the ability to sensitize 721.221-ICP47-A1*002 cells to NK cell lysis. All of the variants tested bound to Mamu-A1*002 (S3A–S3D Fig) and did not affect the cytolytic activity of Mamu-KIR3DL05^-^ NK cells (S3E–S3H Fig). However, several of the peptides did alter Mamu-KIR3DL05^+^ NK cell responses (Fig 3). In the case of Gag GY9, alanine or tryptophan substitutions at position 8 partially (GY9 L8A), or completely (GY9 L8W), abrogated Mamu-KIR3DL05^+^ NK cell inhibition (Fig 3A and 3B). Similar results were obtained for GY9 variants with tyrosine or phenylalanine at this position (S4 Fig), suggesting that the presence of a bulky aromatic side chain at position 8 may interfere with Mamu-A1*002 binding to Mamu-KIR3DL05. In the case of Nef YY9, opposite effects were observed for alanine versus tryptophan substitutions at position 8. Whereas replacement of arginine with alanine (YY9 R8A) further suppressed the cytolytic activity of Mamu-KIR3DL05^+^ NK cells compared to the wild-type peptide, a tryptophan substitution (YY9 R8W) allowed killing to a similar extent as the control peptide that does not bind to Mamu-A1*002 (Fig 3C and 3D). Substitutions at positions 6 and 7 also resulted in partial (YY9 G6A and G6W), or a nearly complete (YY9 I7W), loss of inhibition, suggesting that these residues may also contribute to interactions with Mamu-KIR3DL05. Similar to Gag GY9 and Nef YY9, a valine-to-tryptophan substitution in the penultimate position of Env RY8, in this case position 7 (RY8 V7W), completely abrogated inhibition (Fig 3E and 3F). A significant increase in cytolyic activity was also observed for a tryptophan substitution at position 6 (RY8 R6W) (Fig 3E and 3F). Yet neither alanine nor tryptophan substitutions at position 5 had a detectable effect on Mamu-KIR3DL05^+^ NK cell responses to any of these peptides (Fig 3A–3F). In the case of Vif IW9, single amino acid substitutions were not sufficient to change this disinhibitory peptide into an inhibitory variant. However, a combination of substitutions at positions 8 and 9 (IW9 F8A W9Y) resulted in significant inhibition of Mamu-KIR3DL05^+^ NK cells (Fig 3G and 3H), demonstrating that it is also possible to convert a peptide that permits a cytolytic response into one that suppresses this activity with only a couple of amino acid changes. Taken together, these results reveal a prominent role for residues at the penultimate position of Mamu-A1*002-bound peptides, with additional contributions of the two preceding residues, in modulating Mamu-A1*002 interactions with Mamu-KIR3DL05.

**Fig 4:**
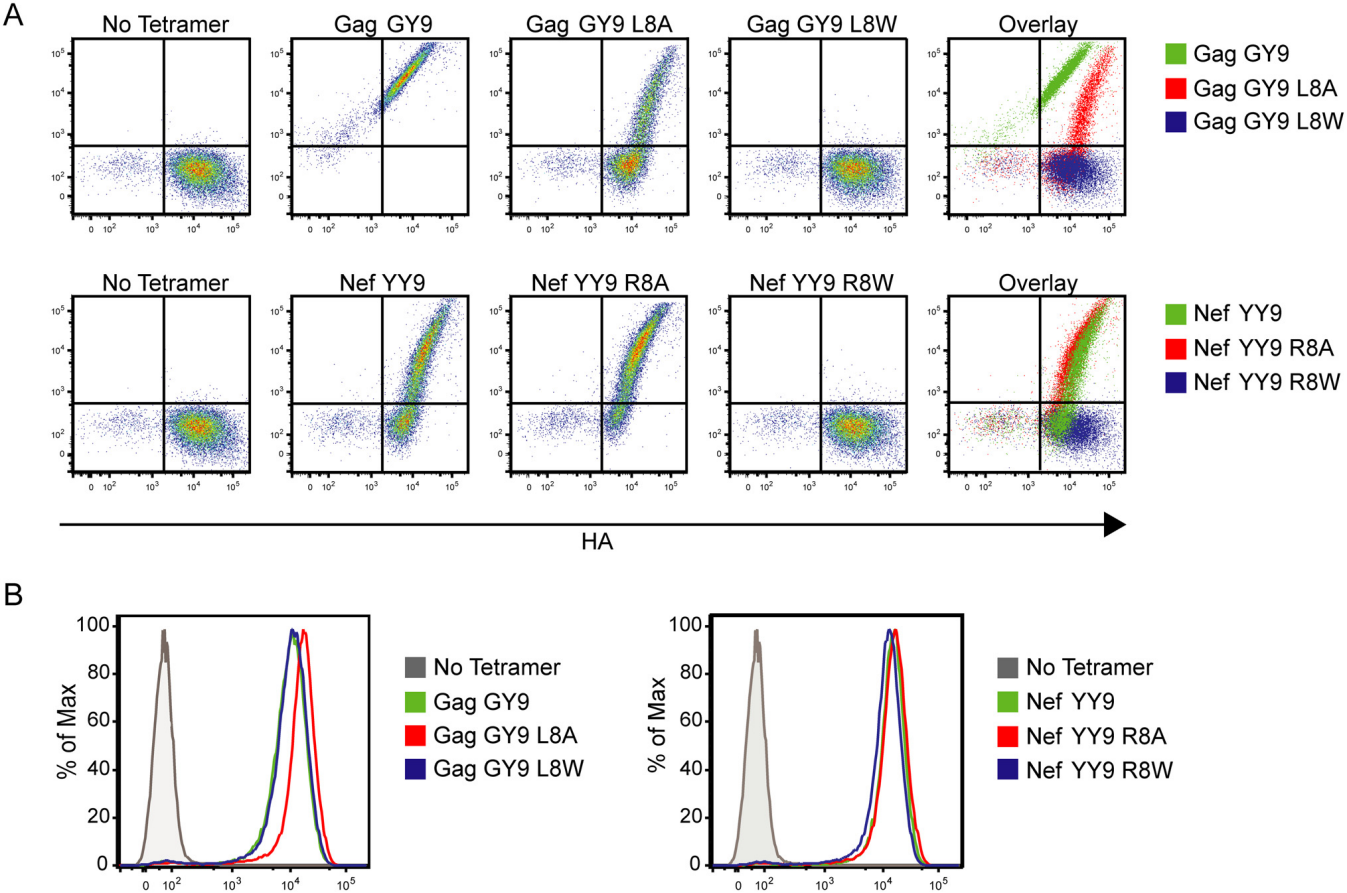
Substitutions at position 8 of Gag GY9 or Nef YY9 alter binding of Mamu-A1*002 to Mamu-KIR3DL05. (A) Jurkat cells expressing HA-tagged Mamu-KIR3DL05 were stained with an anti-HA antibody and Mamu-A1*002 tetramers folded with Gag GY9, Nef YY9, or variant peptides with alanine or tryptophan substitutions at position 8 as indicated. (B) Tetramer integrity was confirmed by staining LILRB1-expressing Ba/F3 cells with each tetramer.

To confirm that these functional differences in NK cell responses reflect the effects of Mamu-A1*002-bound peptides on molecular interactions with Mamu-KIR3DL05, custom Mamu-A1*002 tetramers were synthesized with selected Gag GY9 and Nef YY9 variants and tested for their ability to bind to Mamu-KIR3DL05. Jurkat cells were electroporated with a plasmid DNA construct that expresses HA-tagged Mamu-KIR3DL05 and then stained with tetramer and an HA-specific monoclonal antibody (Fig 4A), as previously described [27]. The integrity of each of the tetramers was also verified by staining a cell line that stably expresses LILRB1, which binds to MHC class I molecules folded with β2-microglobulin independently of MHC-bound peptide (Fig 4B) [32–34]. A comparison of tetramer- versus HA-staining on transfected Jurkat cells revealed that the effects of peptides on Mamu-A1*002 binding to Mamu-KIR3DL05 directly mirrors their effects on cytolytic activity. Just as Gag GY9 L8A partially increases target cell lysis by Mamu-KIR3DL05^+^ NK cells (Fig 3A and 3B), this peptide diminishes Mamu-A1*002 binding to Mamu-KIR3DL05 (shifts tetramer staining to higher levels of Mamu-KIR3DL05/HA on the cell surface) (Fig 4A). Conversely, the Nef YY9 R8A variant, which enhances Mamu-KIR3DL05^+^ NK cell inhibition (Fig 3C and 3D), increases Mamu-A1*002 binding to Mamu-KIR3DL05 compared to the wild-type Nef YY9 peptide (shifts tetramer staining to slightly lower surface levels of Mamu-KIR3DL05/HA) (Fig 4A). Furthermore, the Gag GY9 L8W and Nef YY9 R8W variants, which allow full cytolytic activation of Mamu-KIR3DL05^+^ NK cells (Fig 3A–3D), completely abrogate Mamu-A1*002 binding to Mamu-KIR3DL05 (Fig 4A). These tryptophan substitutions do not interfere with the folding of Mamu-A1*002-peptide complexes, since Gag GY9 L8W and Nef YY9 R8W tetramers retain the ability to bind to LILRB1 (Fig 4B). Thus, the functional effects of amino acid changes at position 8 of Gag GY9 and Nef YY9 on Mamu-KIR3DL05^+^ NK cell responses correspond to their effects on Mamu-A1*002 binding to Mamu-KIR3DL05.

### Structural modeling of amino acid replacements in SIV peptides bound by Mamu-A1*002

**Fig 5:**
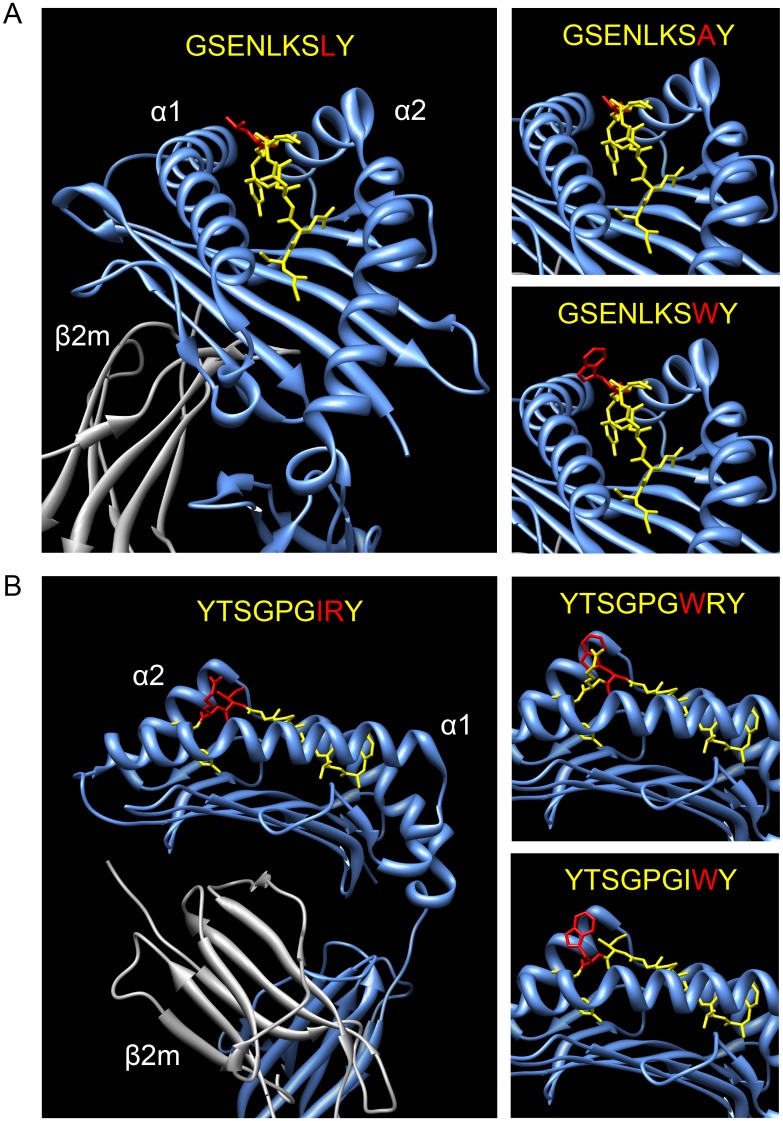
Modeling of amino acid changes in SIV peptides bound by Mamu-A1*002. Three-dimensional crystal structures of Mamu-A1*002 in complex with Gag GY9 (A) and Nef YY9 (B) are shown with alanine or tryptophan substitutions modeled at the positions indicated in red [35,36]. The α1- and α2-domains of the MHC class I heavy chain (blue), β2-microglobulin (grey), and SIV peptide (yellow) are depicted.

Three-dimensional crystal structures of Mamu-A1*002 in complex with Gag GY9 and Nef YY9 reveal how changes in C-terminal positions of these peptides may affect interactions with Mamu-KIR3DL05 [35,36]. These structures show that the side chains of the residues at position 8 of both peptides project over the α1-domain of Mamu-A1*002 in an orientation to interact with Mamu-KIR3DL05 [35,36]. In the case of Gag GY9 L8A, the methyl group of the alanine at this position should not interfere with Mamu-A1*002 binding to Mamu-KIR3DL05 (Fig 5A). However, this side chain is also unlikely to contribute to the stability of the interaction to the same extent as the longer aliphatic side chain of leucine, which probably explains the decrease in tetramer binding (Fig 4A) and the partial increase in cytolytic activity for this peptide (Fig 3A). In the case of Gag GY9 L8W, the bulky indole ring of tryptophan at position 8 projects out of the peptide-binding pocket in an orientation to directly interfere with binding to Mamu-KIR3DL05. Indeed, Mamu-A1*002 tetramer folded with this peptide failed to bind to Mamu-KIR3DL05 under conditions of protein over-expression in transfected cells (Fig 4A). Disruption of Mamu-KIR3DL05 binding to Mamu-A1*002 therefore explains the complete loss of NK cell inhibition and the strong cytolytic response against 721.221-ICP47-A1*002 cells pulsed with this peptide (Fig 3A). Similar modeling of Nef YY9 I7W and R8W in complex with Mamu-A1*002 reveals that the ring structures at positions 7 and 8 of these peptides protrude from the peptide-binding pocket (Fig 5B). Hence, these tryptophan substitutions may also hinder Mamu-KIR3DL05 binding to Mamu-A1*002, accounting for the lack of Mamu-A1*002-YY9 R8W tetramer binding to Mamu-KIR3DL05 (Fig 4A) and the robust cytolytic activity against cells pulsed with each of these peptide variants (Fig 3C).

### Inhibitory peptides dominate over variants that do not suppress NK cell responses

**Fig 6:**
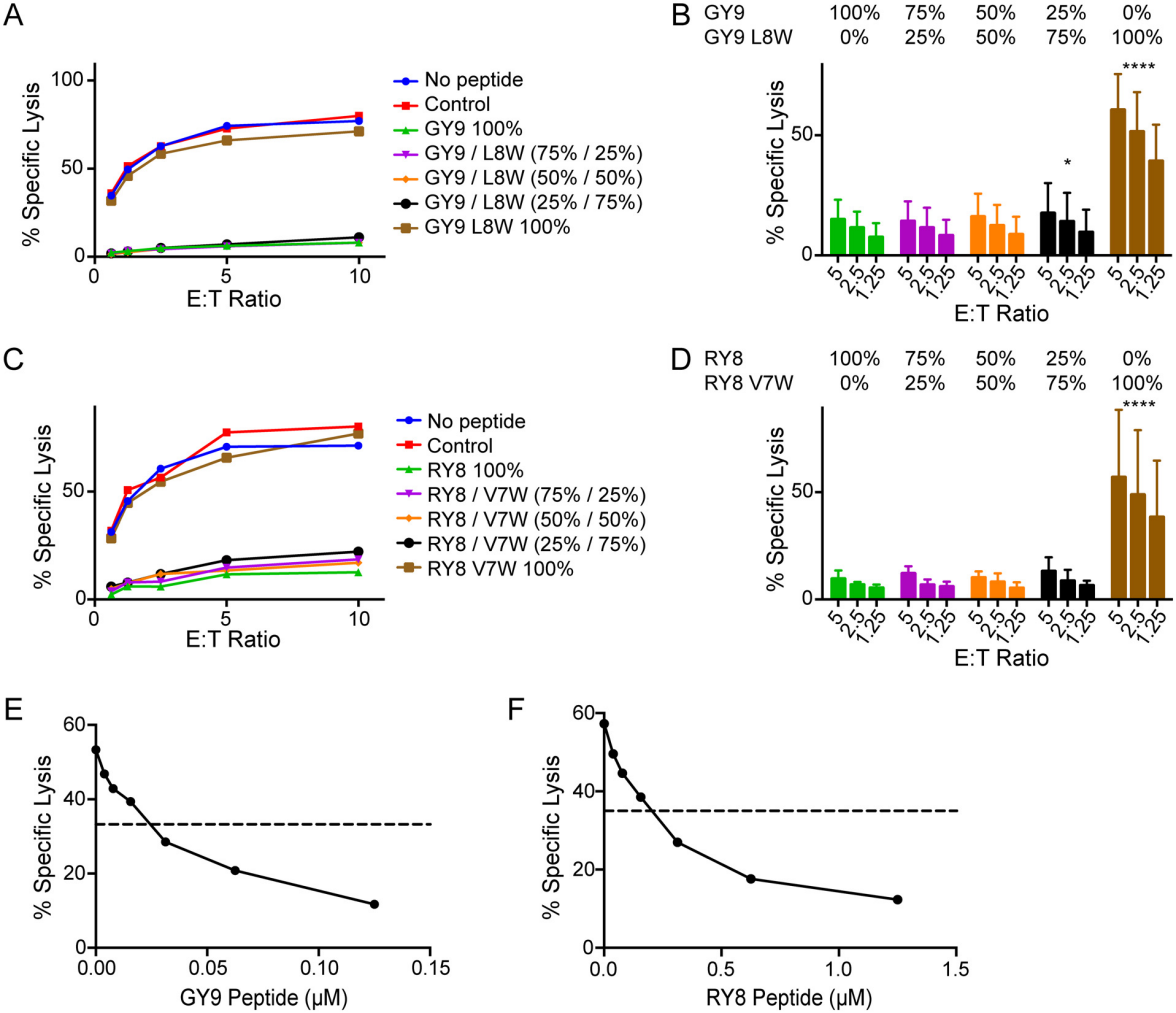
Signals from inhibitory peptides dominate to suppress NK cell activation. 721.221-ICP47-A1*002 cells were pulsed with mixtures of Gag GY9 and GY9 L8W (A, B & E) or Env RY8 and RY8 V7W (C, D & F) and tested for susceptibility to killing by Mamu-KIR3DL05^+^ NK cells in CAM cytotoxicity assays. Representative data (A & C) and mean percent specific lysis (B & D) are shown for three independent experiments using NK cells from different animals. In panels A-D, the percentages of inhibitory versus disinhibitory peptides varied, keeping the total peptide concentration constant at 0.5 μM for GY9/GY9 L8W and 5 μM for RY8/RY8 V7W. Error bars indicate +1 SD and asterisks indicate significant differences in the lysis of target cells pulsed with GY9 or RY8 compared to target cells pulsed with the indicated peptide mixtures (*p<0.05, ****p<0.001 by two-way ANOVA with Dunnett’s test). In panels E-F, target cells were pulsed with increasing concentrations of Gag GY9 (E) or Env RY8 (F) in combination with a fixed concentration of their respective disinhibitory variants (0.375 μM GY9 L8W or 3.75 μM RY8 V7W). The dashed line indicates 50% inhibition where GY9 or RY8 alone defines 100% inhibition and GY9 L8W or RY8 V7W alone defines 0% inhibition. Mamu-A1*002 stabilization on the surface of 721.221-ICP47-A1*002 cells was verified by flow cytometry using the MHC class I-specific monoclonal antibody W6/32 (S5 Fig).

MHC class I molecules present a diverse repertoire of peptides derived from viral and cellular antigens on the surface of virus-infected cells, of which some may stabilize and some may disrupt interactions with any given KIR. We therefore asked which signal dominates when a mixture of peptides that do or do not suppress NK cell activity is presented on the cell surface. 721.221-ICP47-A1*002 cells were pulsed with mixtures of Gag GY9 and GY9 L8W or Env RY8 and RY8 V7W at concentrations that did not over-saturate binding to Mamu-A1*002 (S5A Fig), and were tested for susceptibility to lysis by Mamu-KIR3DL05^+^ NK cells. Cell surface stabilization of Mamu-A1*002 was similar for GY9 versus GY9 L8W and RY8 versus RY8 V7W individually (S5A Fig), or in combination (S5B Fig), and none of the peptide mixtures affected Mamu-KIR3DL05^-^ NK cell responses (S5C Fig). At 25% of the total peptide concentration, Gag GY9 and Env RY8 dominated over their respective disinhibitory variants to fully suppress the cytolytic activity of Mamu-KIR3DL05^+^ NK cells (Fig 6A–6D). To determine the threshold at which the inhibitory effects of these peptides are observed, increasing concentrations of Gag GY9 and Env RY8 were tested in the presence of a fixed, sub-saturating concentration of Gag GY9 L8W and Env RY8 V7W. Half-maximal inhibition of killing was achieved at approximately 5% of the total peptide concentration for both Gag GY9 and Env RY8 (Fig 6E and 6F). Thus, under conditions where inhibitory peptides and their corresponding disinhibitory variants are presented simultaneously by Mamu-A1*002, the inhibitory peptides dominate to suppress Mamu-KIR3DL05^+^ NK cell responses.

### Mamu-KIR3DL05^+^ NK cells are impaired in their ability to suppress SIV replication in Mamu-A1*002^+^ lymphocytes

**Fig 7:**
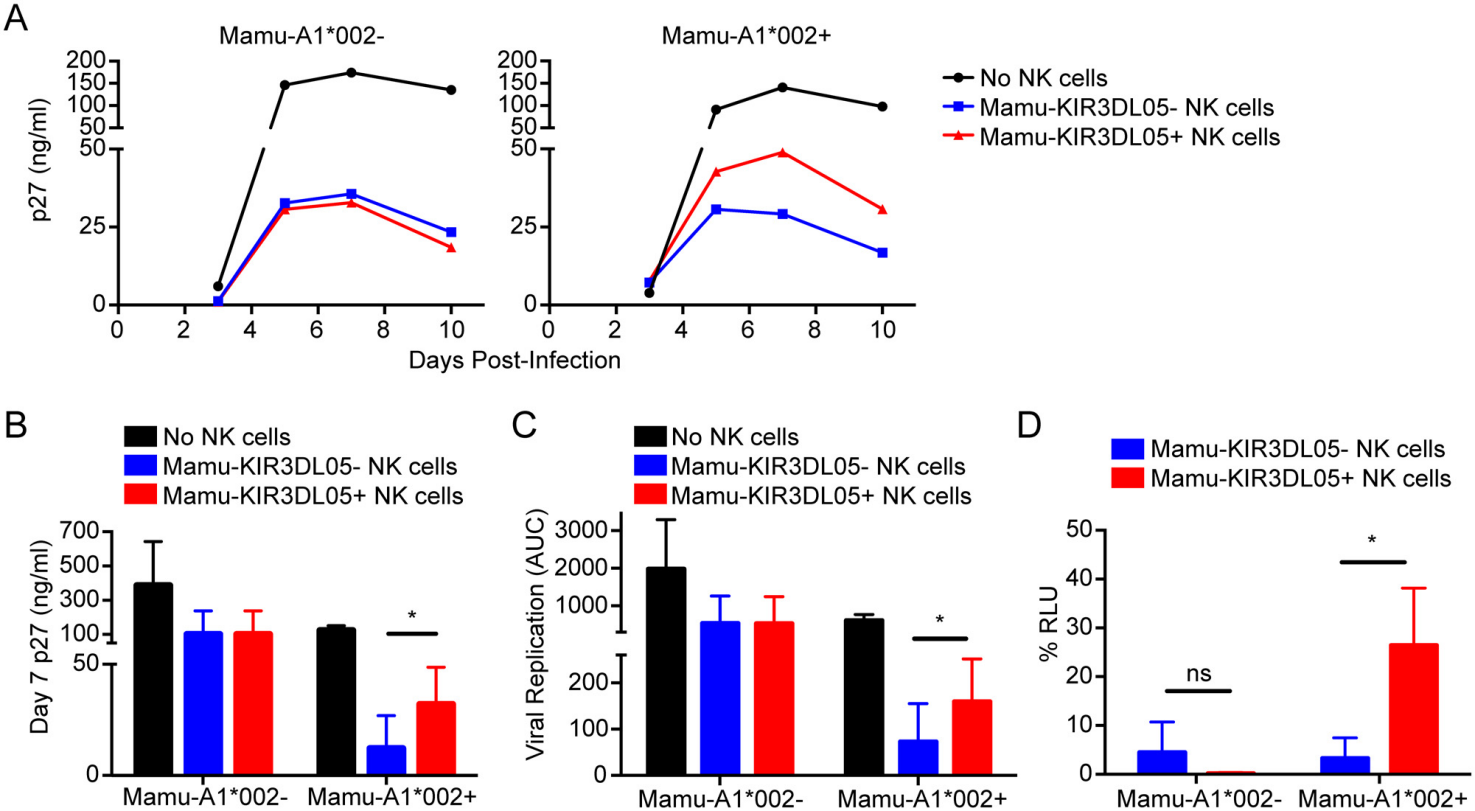
Mamu-KIR3DL05^+^ NK cells exhibit a reduced capacity to suppress SIV replication in cells expressing Mamu-A1*002. (A) CD4^+^ lymphocytes from Mamu-A1*002^-^ versus-A1*002^+^ rhesus macaques were infected with SIV, and incubated either in the absence of NK cells, or in the presence of Mamu-KIR3DL05^+^ or -KIR3DL05^-^ NK cells from the same animal. SIV replication was assessed by measuring the accumulation of SIV p27 in the cell culture supernatant by antigen-capture ELISA. Replication curves are representative of experiments with cells from three different animals. Differences in peak (B) and total (C) SIV replication are based on average p27 concentrations on day 7 post-infection and area under the curve (AUC) calculations, respectively, for three independent experiments. (D) Mamu-KIR3DL05^+^ and -KIR3DL05^-^ NK cells were compared for the ability to lyse Mamu-A1*002^+^ versus-A1*002^-^ target cells infected with SIV. Mamu-A1*002^+^ and -A1*002^-^ 721.221 cell lines were engineered for constitutive expression of CD4, and Tat-inducible expression of a luciferase reporter gene, and infected with SIV for 4 days before incubation with Mamu-KIR3DL05^+^ and -KIR3DL05^-^ NK cells from the same animal in an 8-hour assay. Loss of relative light units (RLU) indicates lysis of luciferase-expressing cells. Percent RLU were calculated from the luciferase activity remaining in virus-infected cells incubated with NK cells relative to maximal luciferase activity in virus-infected cells incubated without NK cells. Error bars indicate + 1 SD of the mean for three separate experiments using NK cells from different animals (*p<0.05 by paired t-test; ns = non-significant).

To determine if the presentation of viral epitopes by Mamu-A1*002 impairs the antiviral activity of Mamu-KIR3DL05^+^ NK cells, SIV replication was compared in Mamu-A1*002^+^ and -A1*002^-^ CD4^+^ lymphocytes co-cultured with Mamu-KIR3DL05^+^ versus-KIR3DL05^-^ NK cells from the same animal. Both NK cell subsets suppressed virus replication to a similar extent in CD4^+^ lymphocytes from Mamu-A1*002^-^ animals (Fig 7A). However, virus replication was consistently higher in the presence of Mamu-KIR3DL05^+^ than-KIR3DL05^-^ NK cells in Mamu-A1*002^+^ CD4^+^ lymphocytes (Fig 7A). A composite analysis of data obtained using cells derived from three different animals revealed significantly higher peak (p = 0.0370, paired t-test) and total (p = 0.0102, paired t-test) virus replication in Mamu-A1*002^+^ CD4^+^ lymphocytes cultured with Mamu-KIR3DL05^+^ NK cells than in those cultured with Mamu-KIR3DL05^-^ NK cells (Fig 7B and 7C). Similar results were also obtained for a comparison of Mamu-KIR3DL05^+^ versus-KIR3DL05^-^ NK cell lysis of SIV-infected 721.221 cell lines that do, or do not, express Mamu-A1*002. Whereas the killing of virus-infected Mamu-A1*002^-^ cells was equivalent for both NK cell subsets, the lysis of SIV-infected Mamu-A1*002^+^ cells by Mamu-KIR3DL05^+^ NK cells was significantly inhibited compared to Mamu-KIR3DL05^-^ NK cells (p = 0.0347, paired t-test) (Fig 7D). These differences in the ability of Mamu-KIR3DL05^+^ versus-KIR3DL05^-^ NK cells to suppress SIV replication and to lyse virus-infected cells are therefore consistent with the specific impairment of Mamu-KIR3DL05^+^ NK cells by inhibitory viral peptides presented on the surface of SIV-infected cells by Mamu-A1*002.

## Discussion

Genetic studies have associated certain combinations of *KIR* and *HLA class I* alleles with the control of HIV, HPV, and HCV infections, suggesting that KIR-MHC class I interactions play an important role in the immune response to these viruses [8,9,13,14]. However, less is known about whether viral peptides affect the interaction of KIR with their ligands during infection, despite abundant evidence demonstrating that KIR recognition is influenced by MHC class I-bound peptides [24–27,32,37], including variants of some HIV peptides [38–40]. In this study, we demonstrate that over a third of the SIV peptides bound by Mamu-A1*002, including the four peptides with the highest affinity for this MHC class I molecule, suppress the cytolytic activity of Mamu-KIR3DL05^+^ NK cells. These peptides therefore either stabilize, or do not interfere with, the recognition of Mamu-A1*002 by Mamu-KIR3DL05. We also found that variants of inhibitory peptides with substitutions at C-terminal positions, particularly the introduction of bulky aromatic residues at the penultimate position, no longer suppress Mamu-KIR3DL05^+^ NK cell lysis of Mamu-A1*002^+^ cells. When inhibitory and disinhibitory peptides are both presented on the surface of Mamu-A1*002^+^ cells, signals from inhibitory peptides overwhelmingly dominate to suppress Mamu-KIR3DL05^+^ NK cell responses. Accordingly, Mamu-KIR3DL05^+^ NK cells were less effective at suppressing viral replication in autologous Mamu-A1*002^+^ CD4^+^ lymphocytes and killing SIV-infected Mamu-A1*002^+^ cells than their Mamu-KIR3DL05^-^ counterparts. These results suggest that viral peptides that promote the recognition of MHC class I ligands by inhibitory KIRs may reduce NK cell lysis of virus-infected cells as a mechanism of immune evasion.

We previously demonstrated that Mamu-A1*002 in complex with certain SIV peptides binds with unusually high avidity to Mamu-KIR3DL05 [27]. Direct staining of unstimulated primary macaque NK cells was observed for Mamu-A1*002 tetramers folded with either Gag GY9 or Env RY8 [27]. This contrasts with human KIRs for which tetramer staining is generally only detectable upon KIR overexpression in transfected cell lines or in activated NK cell clones [25,38,41]. The stabilization of Mamu-A1*002-KIR3DL05 interactions by these viral peptides may be important for the resistance of SIV-infected cells to Mamu-KIR3DL05^+^ NK cells under conditions of incomplete Mamu-A1*002 downregulation by Nef. Similar to the selective downregulation of HLA-A and -B molecules (but not HLA-C molecules) by HIV-1 Nef [4–6], all Mamu-A molecules, and some Mamu-B molecules, are downregulated by the SIV Nef protein [42]. Although MHC class I downmodulation increases the resistance of virus-infected cells to CD8^+^ T cells, decreased surface expression of ligands for inhibitory KIRs can increase their susceptibility to NK cells [6]. Thus, viral epitopes, such as Gag GY9 and Env RY8, that stabilize particularly high avidity MHC class I interactions with inhibitory KIRs may compensate for partial MHC class I downmodulation by Nef to protect virus-infected cells from elimination by NK cells.

The specific residues of SIV peptides bound by Mamu-A1*002 that modulate interactions with Mamu-KIR3DL05 were identified by testing peptide variants in cytotoxicity assays. Although changes at the penultimate position (position 7 of Env RY8 or position 8 of Gag GY9 and Nef YY9) had the most consistent impact on Mamu-KIR3DL05^+^ NK cell responses, changes at the preceding positions of Nef YY9 (positions 6 and 7) and Env RY8 (position 6) also affected cytolytic activity. Whereas partial effects were observed for alanine substitutions, tryptophan substitutions resulted in a complete, or nearly complete, loss of Mamu-KIR3DL05^+^ NK cell inhibition.

Three-dimensional crystal structures of Mamu-A1*002 in complex with Gag GY9 and Nef YY9 suggest possible explanations for these effects [35,36]. In the case of Gag GY9 L8A, the short methyl group of alanine at position 8 is unlikely to stabilize interactions with Mamu-KIR3DL05 as well as the longer aliphatic side chain of leucine, which may account for the partial increase in cytolytic activity against this peptide variant and the decrease in avidity of Mamu-KIR3DL05 for Mamu-A1*002 tetramer folded with this peptide. In contrast, the bulky indole ring of tryptophan at position 8 of Gag GY9 L8W, and at positions 7 and 8 of Nef YY9 I7W and R8W, projects out of the peptide-binding pocket in an orientation predicted to interfere with engagement by Mamu-KIR3DL05. In each case, disruption of Mamu-A1*002 binding to Mamu-KIR3DL05 may satisfy “missing self” through the loss of inhibitory signals from this ligand resulting in full cytolytic activity against these peptide variants and absence of binding of Mamu-KIR3DL05 by Mamu-A1*002 tetramers folded with GY9 L8W and YY9 R8W. A similar mechanism probably explains the inability of Vif IW9 to inhibit Mamu-KIR3DL05^+^ NK cell responses. Vif IW9 naturally contains a bulky phenylalanine residue at position 8, and consistent with the inability of Mamu-A1*002 tetramers folded with this peptide to bind to Mamu-KIR3DL05, even under conditions of KIR overexpression in transfected cells [27], Vif IW9 resulted in robust KIR3DL05^+^ NK cell activity against Mamu-A1*002^+^ target cells. However, replacement of phenylalanine with alanine at position 8, in combination with a tryptophan-to-tyrosine substitution at position 9 (an alternative anchor residue for Mamu-A1*002) [31], resulted in a variant (IW9 F8A W9Y) that partially suppresses Mamu-KIR3DL05^+^ NK activation. Together, these observations illustrate how amino acid changes at C-terminal positions of MHC class I-bound peptides that participate in interactions with inhibitory KIR can modulate NK cell responses, and provide a glimpse into the underlying molecular mechanisms by which these receptors contribute to protective immunity versus immune evasion.

Assays with mixtures of Mamu-A1*002-bound peptides that do or do not inhibit Mamu-KIR3DL05^+^ NK cell responses revealed that signals from inhibitory peptides overwhelmingly dominate to suppress NK cell activation. When present at a concentration of just 5% of a mixture with their respective disinhibitory variants, Gag GY9 and Env RY8 exhibited approximately 50% of their maximal capacity to inhibit the lysis of Mamu-A1*002^+^ target cells. These observations are consistent with the need to prevent NK cell lysis of normal healthy cells presenting diverse self-peptides in complex with MHC class I ligands, and imply that for a viral peptide to cause NK cell degranulation by preventing engagement of an inhibitory KIR, it would need to be presented on the cell surface at a sufficiently high density to overcome negative signals from the same MHC class I ligand loaded with other peptides. In this respect, our results contrast with previous studies, which found that low concentrations of ‘antagonistic’ peptides were sufficient to overcome inhibition by peptides that stabilize HLA-Cw*0102 binding to KIR2DL2 and KIR2DL3 [26,43]. A possible explanation for the difference in our results may be the nature of the peptides examined. Whereas antagonistic peptides are distinguished by weak KIR binding that does not induce NK cell inhibition [26], the peptide variants used in our assays contain tryptophan substitutions with bulky aromatic side chains predicted to abrogate KIR binding. Furthermore, because antagonism is less efficient for high avidity HLA-KIR interactions [26], any antagonistic effects of our variants may have been overcome by the unusually high avidity of Mamu-A1*002-Gag GY9 and -Env RY8 complexes for Mamu-KIR3DL05 [27]. Alternatively, differences in our results may reflect fundamental differences in ligand recognition for KIRs with two versus three immunoglobulin-like domains. In the absence of a D0 domain to stabilize MHC class I binding, 2D KIRs may be more sensitive than 3D KIRs to MHC class I-bound peptides. In support of this possibility, changes at position 8 of HLA class I-bound peptides have been shown to have a more dramatic effect on the K_d_ of HLA-Cw3 binding to KIR2DL2 than on that of HLA-B*57 binding to KIR3DL1 [20,21].

While inhibitory peptide dominance is compatible with the need to prevent NK cell lysis of healthy cells, it also provides an avenue for viruses to gain resistance to NK cells by acquiring changes in epitopes that increase the binding of MHC class I ligands to inhibitory KIRs. Indeed, this may explain the selective pressure for HIV-1 to acquire polymorphisms that increase the binding of KIR2DL2 to virus-infected cells and impair virus suppression by NK cells from KIR2DL2^+^ individuals *in vitro* [19]. Therefore, it is perhaps not a coincidence that the four SIV peptides bound with the highest affinity by Mamu-A1*002, including three immunodominant CD8^+^ T cell epitopes known to be processed and presented on the surface of virus-infected cells (Nef YY9, Env RY8 and Gag GY9), all suppress Mamu-KIR3DL05^+^ NK cell responses. Although multiple factors determine the efficiency of MHC class I presentation, the high affinity of Mamu-A1*002 for these peptides suggests that they may be presented in abundance on the surface of SIV-infected cells expressing this MHC class I molecule. Given that Mamu-KIR3DL05 and -A1*002 are commonly expressed in Indian origin rhesus macaques [27], it is therefore conceivable that SIV may have acquired changes in one or more of these epitopes to stabilize this KIR-MHC class I interaction as a mechanism of NK cell evasion.

In accordance with this possibility, Mamu-KIR3DL05^+^ NK cells were less effective than Mamu-KIR3DL05^-^ NK cells at suppressing SIV replication in autologous CD4^+^ lymphocytes from Mamu-A1*002^+^ macaques. Moreover, the lysis of SIV-infected cells expressing Mamu-A1*002 was also less efficient for Mamu-KIR3DL05^+^ NK cells than for Mamu-KIR3DL05^-^ NK cells. As these differences were not observed using CD4^+^ lymphocytes from Mamu-A1*002^-^ animals, or target cells that lack Mamu-A1*002, these results do not reflect inherent differences in Mamu-KIR3DL05^+^ versus-KIR3DL05^-^ NK cell function. Thus, under physiological conditions of virus infection in primary CD4^+^ lymphocytes and in a stable cell line expressing Mamu-A1*002, Mamu-KIR3DL05^+^ NK cells are specifically impaired in their ability to contain SIV replication.

Similar observations in humans suggest that NK cells exert selective pressure on HIV-1 replication in certain individuals. KIR2DL2-associated HIV-1 polymorphisms were identified in chronically infected individuals that increase the binding of this inhibitory KIR to virus-infected cells and impair the ability of NK cells from KIR2DL2^+^ individuals to suppress virus replication *in vitro* [19]. Although the HLA class I ligands and specific viral peptides associated with these polymorphisms were not identified, subsequent studies defined a few HIV-1 peptides that could enhance the binding of KIR2DL2 to two HLA-C1 allotypes [39,40]. These observations are analogous to the identification of SIV peptides that inhibit Mamu-KIR3DL05^+^ NK cell responses. However, because rhesus macaques do not express KIR2D receptors of the D1-D2 configuration or their HLA-C ligands [44–47], the mechanisms of NK cell inhibition in humans and macaques may differ. Whereas HLA-C is not downregulated by HIV-1 Nef [5,6], Mamu-A1*002 is subject to downregulation by the SIV Nef protein [42]. Thus, it is conceivable that amino acid changes in HIV-1 epitopes that merely eliminate interference with HLA-C binding to KIR2DL2 would be sufficient to inhibit KIR2DL2^+^ NK cells, but substitutions in SIV epitopes that increase the stability of Mamu-KIR3DL05 binding to Mamu-A1*002 may be necessary to overcome incomplete downregulation of this ligand by Nef to adequately suppress KIR3DL05^+^ NK cells.

Although HLA class I-bound peptides also influence interactions with human KIR3DL1 [38,41], functional parallels with Mamu-KIR3DL05 are limited. Whereas Mamu-KIR3DL05^+^ NK cells exhibit impaired NK cell suppression of SIV replication in autologous Mamu-A1*002^+^ lymphocytes, human NK cells from individuals possessing highly-expressed alleles of KIR3DL1 are better able to suppress HIV-1 replication in autologous HLA-B*57^+^ lymphocytes [16]. Moreover, unlike Mamu-KIR3DL05, which appears to be associated with elevated viral loads in SIV-infected animals [48], certain highly expressed alleles of KIR3DL1, in combination with their HLA-Bw4 ligands, are associated with lower viral loads and slower progression to AIDS in HIV-1 infected individuals [9]. These contrary observations may reflect fundamental differences in the molecular interactions of Mamu-KIR3DL05 versus KIR3DL1 with their ligands, as these receptors represent highly divergent products of non-orthologous genes [27]. However, from our limited knowledge of the MHC class I ligands of rhesus macaque KIRs and the effects of viral peptides on KIR-MHC class I interactions in either humans or macaques, it is difficult to generalize these findings to other KIR-MHC class I interactions. Indeed, it is possible that Mamu-KIR3DL05^+^ NK cells may suppress SIV replication in the context of MHC class I ligands other than Mamu-A1*002 that have yet to be defined, or that the presentation of HIV-1 epitopes by some HLA-Bw4 molecules may suppress KIR3DL1^+^ NK cell responses.

In summary, we show that 28 of 75 SIV peptides bound by Mamu-A1*002 inhibit Mamu-KIR3DL05^+^ NK cell responses, that changes at C-terminal positions of these peptides differentially affect NK cell activity, and that signals from inhibitory peptides dominate over disinhibitory variants to suppress NK cell lysis. Insights into the molecular basis for these peptide-dependent effects on NK cell responses are provided by modeling amino acid replacements into available three-dimensional structures of SIV Gag GY9 and Nef YY9 in complex with Mamu-A1*002. To our knowledge, this represents the most comprehensive analysis to date of the functional effects of viral peptides on a specific KIR-MHC class I interaction. The identification of multiple viral peptides bound with high affinity by Mamu-A1*002 that enhance binding to Mamu-KIR3DL05 and suppress NK cell activation, together with a deficit in the ability of Mamu-KIR3DL05^+^ NK cells to suppress SIV replication in Mamu-A1*002^+^ lymphocytes, supports a mechanism by which immunodeficiency viruses may reduce their susceptibility to NK cells by acquiring changes in epitopes that stabilize MHC class I interactions with inhibitory KIRs.

## Materials and Methods

### Ethics statement

The Indian origin rhesus macaques (*Macaca mulatta*) used in this study were housed and cared for in an indoor ABSL2+ facility at the New England Primate Research Center (NEPRC) in accordance with standards of the Association for Assessment and Accreditation of Laboratory Animal Care and the Harvard Medical School Animal Care and Use Committee. Animal experiments were approved by the Harvard Medical Area Standing Committee on Animals under protocol 04873 and conducted according to the principles described in the *Guide for the Care and Use of Laboratory Animals* [49]. Steps taken to improve animal welfare included social housing and environmental enrichment such as foraging opportunities and manipulable devices. Water was supplied ad libitum and animals were provided commercial monkey chow twice daily and fresh produce at least three times per week. Animals were monitored twice daily by animal care and veterinary staff, and suffering due to experimental procedures was minimized by sedation with ketamine HCl prior to blood collection.

### Stable cell lines

To produce 721.221-ICP47-A1*002 cells, rhesus macaque Mamu-A1*002 cDNA was cloned into the pQCXIP retroviral vector (Clontech). This vector was cotransfected with pVSV-G (Clontech) into GP2-293 cells and supernatant was harvested two days post-transfection. The supernatant was centrifuged in Ultracel 50k filter centrifuge tubes (Millipore) to yield concentrated VSV-G pseudotyped MLV-based particles. 721.221-ICP47 cells, a derivative of the MHC class I-deficient 721.221 cell line [50] that expresses HSV-1 ICP47 to inhibit the transporter associated with antigen processing (TAP) complex [29,30], were transduced by incubation with concentrated virus for 3 hours at 37°C. Three days later, cells were placed under selection with 0.4 μg/mL puromycin (Invitrogen). 721.221-CD4-A1*002-LTRluc cells were produced by sequential transduction of 721.221 cells with pQCXIP expressing rhesus macaque CD4, a pLNSX-derived retroviral vector containing the firefly luciferase gene under the control of the SIV LTR, and pQCXIH (Clontech) expressing Mamu-A1*002 using a similar procedure. Cells were placed under selection with 0.4 μg/mL puromycin, 500 μg/mL of G418, and 100 μg/mL of hygromycin additively after each transduction.

### NK cell expansion and cell culture

PBMC (5 × 10^6^ cells) were stimulated with 1 × 10^7^ γ-irradiated K562 Clone 9.mbIL21 cells [28] in a volume of 40 mL of NKEM: RPMI 1640 (Invitrogen) supplemented with 10% FBS (Invitrogen), glutamine (Invitrogen), Primocin (InvivoGen), and 50 U/mL IL-2 (NIH AIDS Reagent Program, Division of AIDS, NIAID, NIH, contributed by Dr. Maurice Gately, Hoffmann—La Roche Inc.) [51]. On days 3 and 5 after culture, cells were resuspended in fresh medium. The expanded NK cells were re-stimulated on day 7, and weekly thereafter, with additional γ-irradiated K562 Clone 9.mbIL21 cells at a 1:1 ratio. From day 7 onward, expanded cells were resuspended in fresh medium at 4 × 10^5^ cells/mL 2–3 times weekly.

### NK cell sorting

Expanded NK cell cultures were incubated with anti-CD3 Ab (clone 6G12) and T cells were depleted using pan-mouse IgG Dynabeads (Dynal Biotech). Mamu-KIR3DL05^+^ and -KIR3DL05^-^ subsets were separated by FACS using a Mamu-A1*002 tetramer folded with Gag_71-79_ GY9 that binds Mamu-KIR3DL05 [27]. NK cells were stained with PE-conjugated Mamu-A1*002-GY9 tetramer for 30 minutes at 37°C followed by staining with anti-CD3-Pacific Blue (clone SP34-2; BD Biosciences) and anti-NKG2A-APC (clone Z199; Beckman Coulter) or anti-NKG2A-Pacific Blue and anti-CD3-FITC (clone SP34; BD Biosciences) for 20 minutes at 25°C. Tetramer^+^CD3^-^NKG2A^+^ and Tetramer^-^CD3^-^NKG2A^+^ subsets were sorted using a FACSAria II (BD Biosciences). After sorting, these NK cells subsets were stimulated with γ-irradiated K562 Clone 9.mbIL21 cells and maintained as described above. Mamu-KIR3DL05^+^ and -KIR3DL05^-^ NK cell subsets were sorted from PBMC by the same procedure for immediate use in viral suppression assays.

### Calcein acetoxymethyl ester (CAM) cytotoxicity assay

721.221-ICP47-A1*002 cells were incubated overnight at 26°C with SIV peptides (GenScript and Mimotopes) in Hybridoma-Serum Free Medium (Invitrogen) to stabilize cell surface A1*002-peptide complexes. A GY9 variant with S2A and Y9G substitutions at anchor residues was included as a non-A1*002-binding peptide control. An aliquot of 2 × 10^5^ peptide-pulsed cells were then stained with a PE-conjugated pan-MHC class I specific antibody (clone W6/32; Dako) to verify the surface stabilization of MHC class I molecules. The remaining peptide-pulsed cells were stained with CAM (Invitrogen) at a 1:100 dilution for 1 hour at 26°C. CAM-stained cells were washed and then incubated with Mamu-KIR3DL05^+^ or -KIR3DL05^-^ NK cells for 4 hours at E:T ratios between 0.5:1 and 10:1 in NKEM at 26°C. The release of CAM into the supernatant was measured using a fluorescent plate reader (excitation 485 nm, absorption 530 nm). Percent specific lysis was calculated as (test release—spontaneous release) / (maximum release—spontaneous release).

### Tetramer staining

Ten million Jurkat cells were electroporated with 25 μg of pCGCG-KIR3DL05*008-HA construct or empty pCGCG (250 V, 950 μF) in 400 μl RPMI in a 4 mm cuvette (BioRad) then cultured in 9 ml RPMI supplemented with 10% FBS for 22 hours. One million cells were stained with APC-conjugated Mamu-A1*00201 tetramers (NIH Tetramer Core Facility) folded with either GY9 (GSENLKSLY), GY9 L8A (GSENLKSAY), GY9 L8W (GSENLKSWY), YY9 (YTSGPGIRY), YY9 R8A (YTSGPGIAY), or YY9 R8W (YTSGPGIWY) peptide (Genscript; 30 minutes, 37°C) followed by staining with anti-HA-PE (Miltenyi) for 10 minutes at 4°C. Cells were washed in PBS and fixed in 4% paraformaldehyde. To verify the proper folding of tetramers with peptide variants, 1 × 10^6^ Ba/F3 cells expressing LILRB1 (kindly provided by Simon Kollnberger, University of Oxford) were stained with each tetramer as previously described [32]. All data was acquired using a LSR II flow cytometer (BD Biosciences) and analyzed using FlowJo 8.8.6.

### Structural modeling

Molecular graphics and analyses were performed with the UCSF Chimera package developed by the Resource for Biocomputing, Visualization, and Informatics at the University of California, San Francisco (supported by NIGMS P41-GM103311). Modeling of amino acid substitutions in the structures of Mamu-A1*002 in complex with GY9 (PDB 3JTS) [35] or YY9 (PDB 3JTT) [36] were carried out using the swapaa function in which rotamer position is determined by minimal clash score, maximal hydrogen bonding, and probability according to the Dunbrack rotamer library [52].

### Viral suppression assay

The target cells were prepared by depletion of CD8^+^ cells from PBMC by treatment with CD8-Dynabeads (Dynal Biotech) followed by ConA-activation (5 μg/ml) in RPMI 1640 supplemented with 10% FBS, glutamine, HEPES (Invitrogen), and Primocin (R10) for 4 days. On the day of the assay, 2 × 10^6^ target cells were infected with 100 ng p27 SIV_mac_239 in minimal volume for 3 hours at 37°C, then washed. Mamu-KIR3DL05^+^ and -KIR3DL05^-^ NK cell subsets were sorted as described above and were incubated with 2 × 10^4^ SIV-infected autologous CD4^+^ target cells in R10 + 20 U/mL IL-2 in duplicate wells at a 5:1 or 3:1 E:T ratio. On days 3, 5, 7, and 9/10, 50 uL of supernatant was sampled with replacement. SIV titer was determined by SIV p27 Antigen Capture Assay (Advanced Bioscience Laboratories). Area under the curve was calculated using Prism 6 (GraphPad).

### Luciferase lysis assay

Four days prior to the assay, 721.221-CD4-A1*002-LTRluc cells, which express luciferase when infected with SIV, were infected by spinoculation [53] in the presence of 4 μg/mL of Polybrene (Sigma-Aldrich). On the day of the assay, infected cells were washed three times and 10^4^ infected cells were plated with between 1.5 x 10^4^ and 10^5^ effector cells in 200 μL of NKEM and incubated for 8 hours at 37°C. Luciferase activity was measured using BriteLite Plus (Perkin Elmer). Control wells contained uninfected 721.221-CD4-A1*002-LTRluc cells. % RLU was defined as (RLU infected with NK cells—RLU uninfected with NK cells) / (RLU infected without NK cells—RLU uninfected without NK cells)*100.

## Supporting Information

S1 Fig Stabilization of cell surface Mamu-A1*002 expression by peptide pulse. Stabilization of Mamu-A1*002 on the surface of 721.221-ICP47-A1*002 cells pulsed with the indicated SIVmac239 peptides was determined by staining with the pan-MHC class I monoclonal antibody W6/32 and the relative gMFI normalized to cells incubated without peptide was calculated. Bars represent the mean relative gMFI for two independent experiments and error bars indicate +1 SD.
(TIF)

S2 Fig Lysis of peptide-pulsed cells by Mamu-KIR3DL05^-^ NK cells. Mamu-KIR3DL05^-^ NK cells were incubated at a 5:1 E:T ratio with CAM-labeled 721.221-ICP47-A1*002 target cells pulsed with the indicated SIVmac239 peptides. Percent specific lysis was calculated from the amount of CAM released into the culture supernatant after a 4-hour incubation. Bars represent the mean percent specific lysis for experiments using NK cells from three different animals. Peptides are ordered from highest to lowest affinity for Mamu-A1*002 according to Loffredo et al. [31]. Previously defined CD8^+^ T cell epitopes are indicated by purple bars and controls include target cells incubated without peptide (blue) or with a GY9 variant with substitutions at anchor positions that abrogate binding to Mamu-A1*002 (red). Error bars indicate +1 SD.
(TIF)

S3 Fig Mamu-KIR3DL05^-^ NK cell lysis of cells presenting variant peptides and stabilization of cell surface Mamu-A1*002. (A-D) Stabilization of Mamu-A1*002 on the surface of 721.221-ICP47-A1*002 cells pulsed with the peptide variants indicated was determined by staining with the pan-MHC class I monoclonal antibody W6/32 and the relative gMFI normalized to cells incubated without peptide is shown. Data is representative of three independent experiments. Mamu-KIR3DL05^-^ NK cells were incubated with CAM-labeled 721.221-ICP47-A1*002 target cells pulsed with variants of Gag GY9 (E), Nef YY9 (F), Env RY8 (G), and Vif IW9 (H), and target cell lysis was assessed after 4 hours at the indicated E:T ratios. Data is representative of experiments using NK cells from three different animals.
(TIF)

S4 Fig Abrogation of GY9 inhibitory capacity by aromatic amino acid substitutions at p8. (A) Mamu-KIR3DL05^+^ and -KIR3DL05^-^ NK cells from the same animal were incubated with CAM-labeled 721.221-ICP47-A1*002 target cells pulsed with the indicated variants of GY9. Percent specific lysis was calculated from the amount of CAM released into the culture supernatant after 4 hours at the indicated E:T ratios. The results shown are representative of data obtained with NK cells from three different animals. (B) Bar graphs summarize the mean percent specific lysis for independent experiments with Mamu-KIR3DL05^+^ NK cells from three different animals. Error bars indicate +1 SD and asterisks indicate significant differences in the lysis of target cells pulsed with wild-type GY9 compared to target cells pulsed with specific peptide variants (****p<0.001 by two-way ANOVA with Dunnett’s test). (C) Stabilization of Mamu-A1*002 on the surface of 721.221-ICP47-A1*002 cells was determined by staining with the pan-MHC class I monoclonal antibody W6/32 and the relative gMFI normalized to cells incubated without peptide is shown. Data is representative of three independent experiments.
(TIF)

S5 Fig Mamu-KIR3DL05^-^ NK cell lysis of cells incubated with peptide mixtures and stabilization of cell surface Mamu-A1*002. Stabilization of Mamu-A1*002 on the surface of 721.221-ICP47-A1*002 cells pulsed with serial dilutions of the peptides indicated (A) or the peptide mixtures indicated (B) was determined by staining with the pan-MHC class I monoclonal antibody W6/32. Relative gMFI is normalized to cells incubated without peptide. Data is representative of three independent experiments. (C) 721.221-ICP47-A1*002 cells were pulsed with mixtures of Gag GY9 and GY9 L8W or Env RY8 and RY8 V7W and tested for susceptibility to killing by Mamu-KIR3DL05^-^ NK cells in CAM cytotoxicity assays. Representative data are shown for three independent experiments using NK cells from different animals.
(TIF)
