# Supplementary figures and images for: Suppression of a Natural Killer Cell Response by Simian Immunodeficiency Virus Peptides
Source: PLoS Pathog. 2015 Sep 2;11(9):e1005145. doi: 10.1371/journal.ppat.1005145 (PMC4557930; doi:10.1371/journal.ppat.1005145)

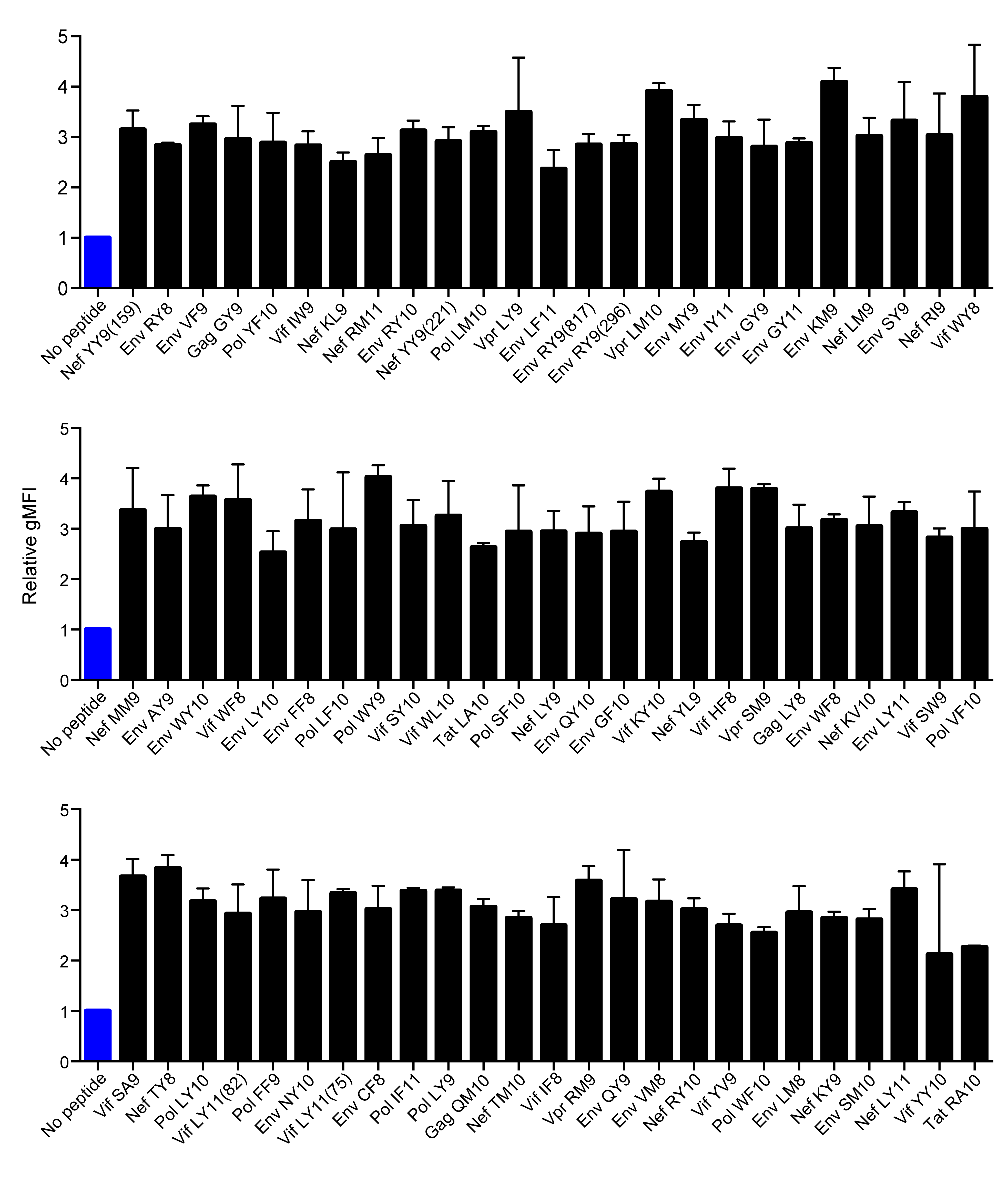

Supplement: S1 Fig — Stabilization of Mamu-A1*002 on the surface of 721.221-ICP47-A1*002 cells pulsed with the indicated SIVmac239 peptides was determined by staining with the pan-MHC class I monoclonal antibody W6/32 and the relative gMFI normalized to cells incubated without peptide was calculated. Bars represent the mean relative gMFI for two independent experiments and error bars indicate +1 SD. (TIF) [file ppat.1005145.s001.tif]

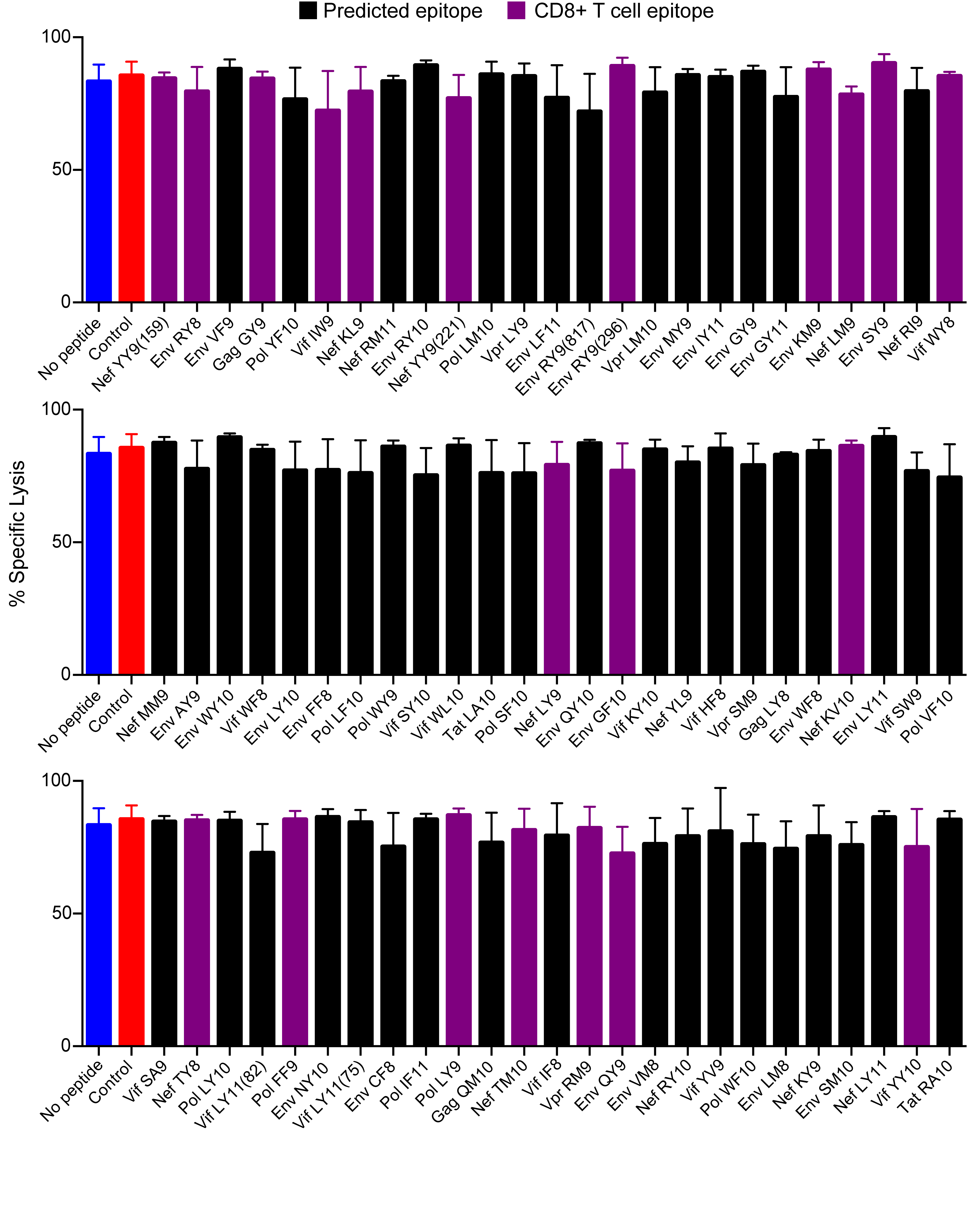

Supplement: S2 Fig — Mamu-KIR3DL05- NK cells were incubated at a 5:1 E:T ratio with CAM-labeled 721.221-ICP47-A1*002 target cells pulsed with the indicated SIVmac239 peptides. Percent specific lysis was calculated from the amount of CAM released into the culture supernatant after a 4-hour incubation. Bars represent the mean percent specific lysis for experiments using NK cells from three different animals. Peptides are ordered from highest to lowest affinity for Mamu-A1*002 according to Loffredo et al. [31]. Previously defined CD8+ T cell epitopes are indicated by purple bars and controls include target cells incubated without peptide (blue) or with a GY9 variant with substitutions at anchor positions that abrogate binding to Mamu-A1*002 (red). Error bars indicate +1 SD. (TIF) [file ppat.1005145.s002.tif]

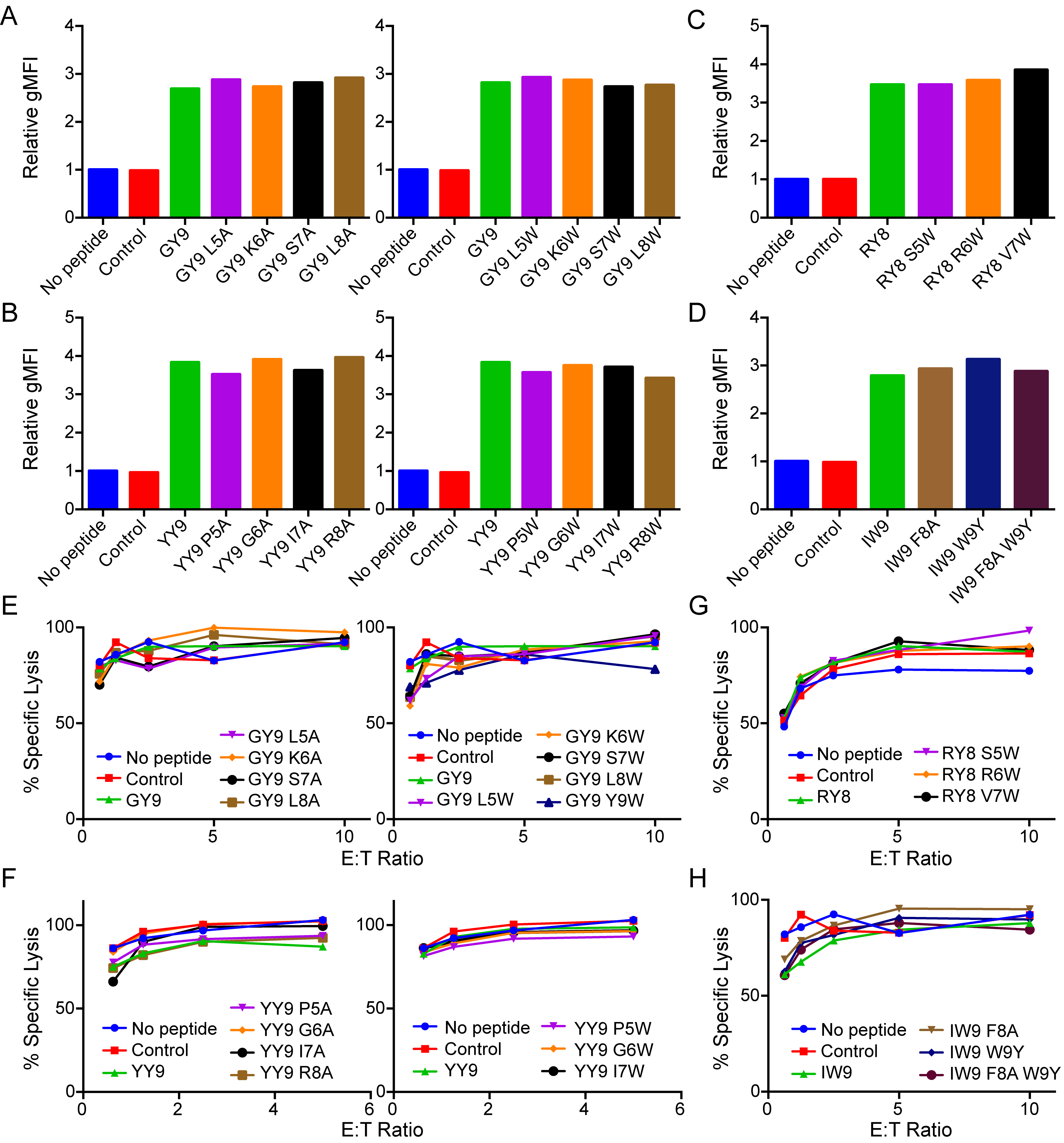

Supplement: S3 Fig — (A-D) Stabilization of Mamu-A1*002 on the surface of 721.221-ICP47-A1*002 cells pulsed with the peptide variants indicated was determined by staining with the pan-MHC class I monoclonal antibody W6/32 and the relative gMFI normalized to cells incubated without peptide is shown. Data is representative of three independent experiments. Mamu-KIR3DL05- NK cells were incubated with CAM-labeled 721.221-ICP47-A1*002 target cells pulsed with variants of Gag GY9 (E), Nef YY9 (F), Env RY8 (G), and Vif IW9 (H), and target cell lysis was assessed after 4 hours at the indicated E:T ratios. Data is representative of experiments using NK cells from three different animals. (TIF) [file ppat.1005145.s003.tif]

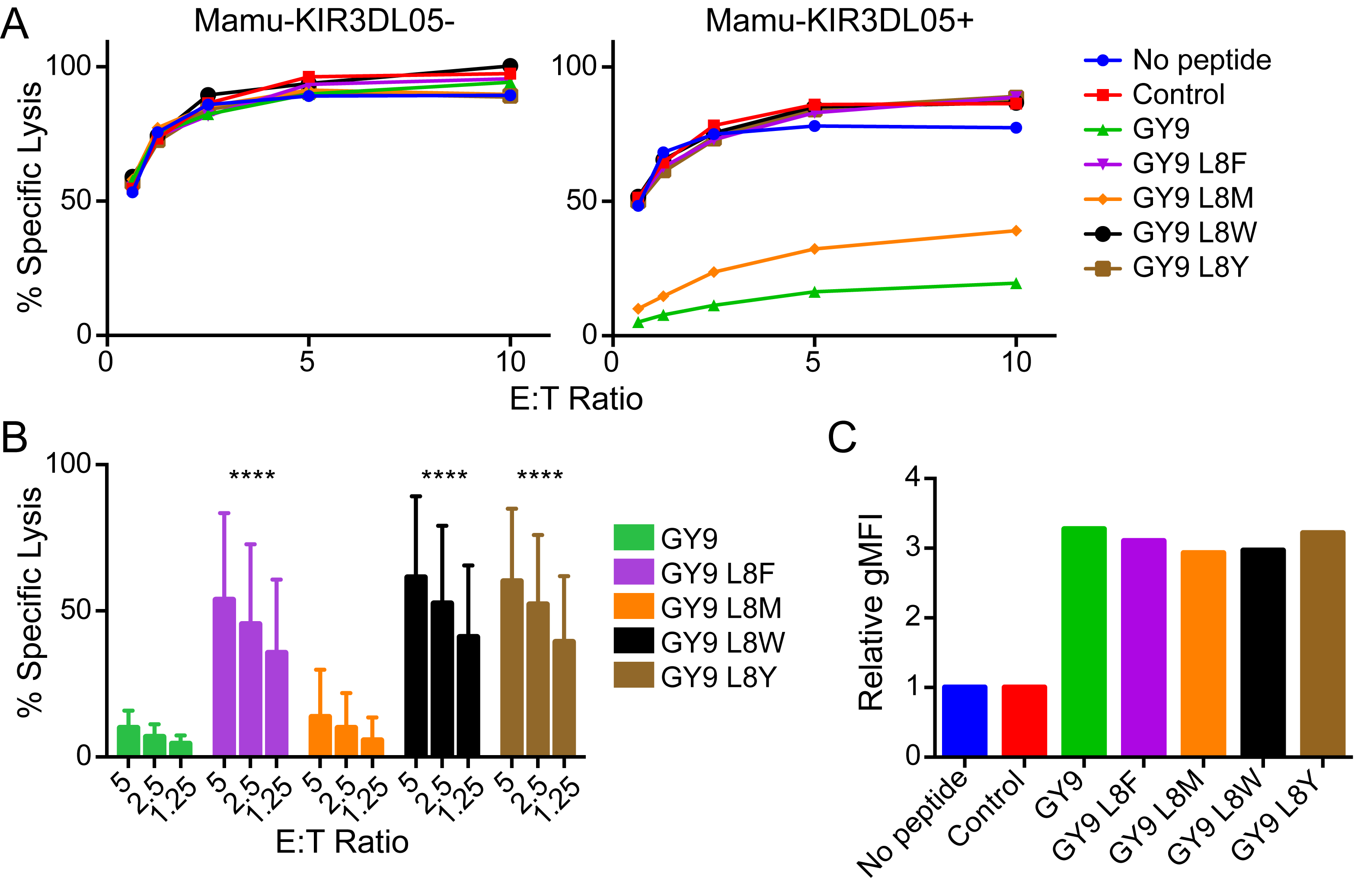

Supplement: S4 Fig — (A) Mamu-KIR3DL05+ and -KIR3DL05- NK cells from the same animal were incubated with CAM-labeled 721.221-ICP47-A1*002 target cells pulsed with the indicated variants of GY9. Percent specific lysis was calculated from the amount of CAM released into the culture supernatant after 4 hours at the indicated E:T ratios. The results shown are representative of data obtained with NK cells from three different animals. (B) Bar graphs summarize the mean percent specific lysis for independent experiments with Mamu-KIR3DL05+ NK cells from three different animals. Error bars indicate +1 SD and asterisks indicate significant differences in the lysis of target cells pulsed with wild-type GY9 compared to target cells pulsed with specific peptide variants (****p<0.001 by two-way ANOVA with Dunnett’s test). (C) Stabilization of Mamu-A1*002 on the surface of 721.221-ICP47-A1*002 cells was determined by staining with the pan-MHC class I monoclonal antibody W6/32 and the relative gMFI normalized to cells incubated without peptide is shown. Data is representative of three independent experiments. (TIF) [file ppat.1005145.s004.tif]

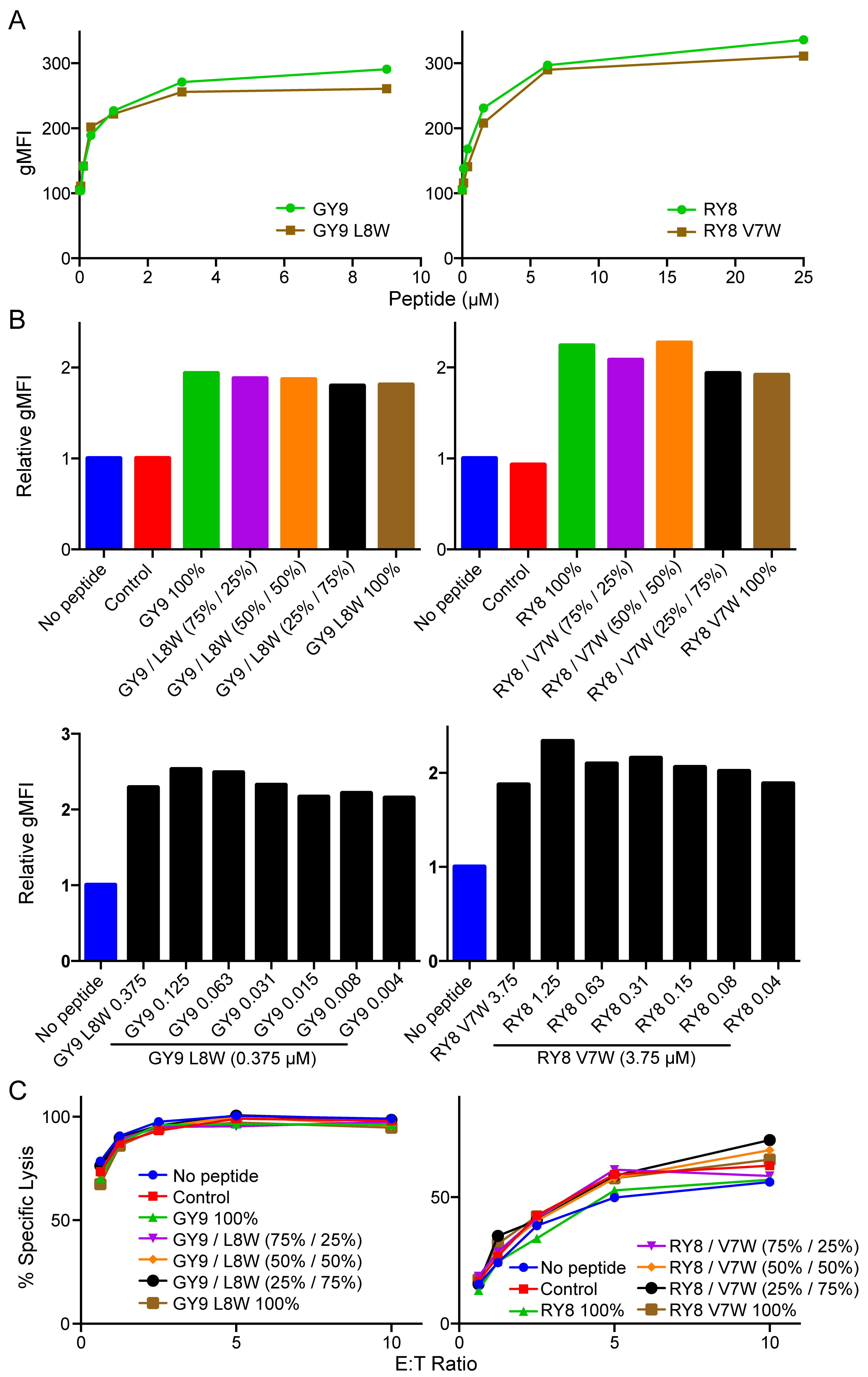

Supplement: S5 Fig — Stabilization of Mamu-A1*002 on the surface of 721.221-ICP47-A1*002 cells pulsed with serial dilutions of the peptides indicated (A) or the peptide mixtures indicated (B) was determined by staining with the pan-MHC class I monoclonal antibody W6/32. Relative gMFI is normalized to cells incubated without peptide. Data is representative of three independent experiments. (C) 721.221-ICP47-A1*002 cells were pulsed with mixtures of Gag GY9 and GY9 L8W or Env RY8 and RY8 V7W and tested for susceptibility to killing by Mamu-KIR3DL05- NK cells in CAM cytotoxicity assays. Representative data are shown for three independent experiments using NK cells from different animals. (TIF) [file ppat.1005145.s005.tif]
